# Biochemical and functional characterization of *Porphyromonas gingivalis* HmuS protein reveals its participation in heme metabolism

**DOI:** 10.3389/fmicb.2026.1788076

**Published:** 2026-05-19

**Authors:** Patryk Cierpisz, Michał Śmiga, Michał Tracz, Ronivaldo Rodrigues da Silva, Jennifer L. DuBois, Teresa Olczak

**Affiliations:** 1 Laboratory of Medical Biology, Faculty of Biotechnology, University of Wrocław, Wrocław, Poland; 2 Laboratory of Mass Spectrometry, Faculty of Biotechnology, University of Wrocław, Wrocław, Poland; 3 Department of Chemistry and Biochemistry, Montana State University, Bozeman, MT, United States

**Keywords:** chelatase, heme metabolism, HmuS, iron acquisition, periodontal diseases, *Porphyromonas gingivalis*

## Abstract

**Background:**

*Porphyromonas gingivalis* lacks the complete heme biosynthesis pathway and does not utilize siderophores to acquire iron. The exact mechanism of iron extraction from heme in this bacterium is unknown. This study aimed to characterize the *P. gingivalis* HmuS^Pg^ protein and to compare its properties with those of the *Bacteroides fragilis* homologous protein.

**Methods:**

UV-visible spectroscopy was used to demonstrate heme binding and follow heme metabolism. Porphyrin levels in bacterial cells and pigment were determined using HPLC or LC-MS, and iron content using atomic absorption spectroscopy or the ferrozine-based method. The influence of the *hmuS^Pg^* gene deletion on the expression of other genes was analyzed using RNA-seq, RT-qPCR, Western blotting, and enzymatic assay.

**Results:**

Although the HmuS^Pg^ contains a domain characteristic of cobaltochelatases and therefore may be assigned to the I class chelatase family, the protein exhibits distinct features. HmuS^Pg^ is a large protein composed of 6 domains and possesses, predicted by structural modeling, two potential heme-binding sites. *In vitro*, HmuS^Pg^ binds heme. NADH and the inner membrane fraction of *P. gingivalis* stimulate its *in vitro* engagement in heme metabolism. A significantly smaller amount of PPIX, determined in bacterial cells grown in liquid media and in the pigment deposited on the bacterial cells of the Δ*hmuS^Pg^* mutant strain, suggests it may have lost the ability to release iron from heme and produce PPIX. Inactivation of the *hmuS^Pg^* gene results in reduced biofilm formation and induces changes in the expression of genes involved in heme uptake and homeostasis.

**Conclusion:**

HmuS^Pg^ may be used in heme metabolism under certain *in vivo* circumstances, where heme is still available, iron is efficiently sequestered by host proteins, and intracellular iron reserves are depleted.

## 1 Introduction

Periodontal diseases are a group of infectious inflammatory diseases that destroy tooth-supporting tissues, cause gum bleeding, and eventually cause tooth loss (Hajishengallis, 2015; Tonetti et al., 2018; Chen et al., 2021; Hajishengallis and Lamont, 2021; Nascimento et al., 2024). One of the main etiological agents and keystone pathogens participating in the onset and progression of periodontitis is the Gram-negative anaerobic bacterium, *Porphyromonas gingivalis* (Hajishengallis et al., 2012; Hajishengallis and Lamont, 2012). Iron and heme are crucial for pathogenic bacteria living in the human host, but they are not available in free form due to their binding by iron- and heme-sequestering proteins (Śmiga and Olczak, 2025a). Like many members of the Bacteroidota phylum, *P. gingivalis* is a heme auxotroph lacking the complete heme biosynthesis pathway (Roper et al., 2000; Kusaba et al., 2002; Nelson et al., 2003; Rocha et al., 2019). *P. gingivalis* encodes only four homologous proteins to those of the final steps of the protoporphyrin IX (PPIX)-dependent heme biosynthesis pathway: uroporphyrinogen III synthase (HemD), coproporphyrinogen III oxidase (HemN), protoporphyrinogen IX dehydrogenase (HemG), and ferrochelatase (HemH). Although *P. gingivalis* requires heme for growth, supplementation of the culture medium with PPIX and inorganic iron effectively supports its proliferation. (Olczak et al., 2012; Gao et al., 2010, 2018; Śmiga et al., 2024a,b). One of the explanations of this process is the hypothesis that heme can be formed from PPIX and inorganic iron due to preserved HemH ferrochelatase, a terminal enzyme of the heme biosynthesis pathway that catalyzes the insertion of ferrous iron into the PPIX ring (Śmiga et al., 2026).

A typical heme uptake system of Gram-negative bacteria is the heme uptake (Hmu) system of *Yersinia pestis* (HmuRSTUV) (Thompson et al., 1999; Hornung et al., 1996) or the heme uptake (Hem) system of *Yersinia enterocolitica* (Stojiljkovic and Hantke, 1992). Heme is transported from the external environment across the outer membrane through TonB-dependent outer membrane receptors (TDRs) (e.g., *Y. enterocolitica* HmuR or *Y. pestis* HemR), powered by electrochemical potential, proton motive force delivered by the TonB/ExbB/ExbD protein complex (Higgs et al., 1998, 2002; Ferguson et al., 2007; Noinaj et al., 2010; Silale and van den Berg, 2023). Depending on the bacterium, TDRs recognize hemoglobin, haptoglobin-hemoglobin, or heme transferred by hemophores or hemophore-like proteins and then transport heme into the periplasmic space (Burkhard and Wilks, 2007; Ascenzi et al., 2015). Further, heme is shuttled by periplasmic binding proteins (e.g., *Y. pestis* HmuT or *Y. enterocolitica* HemT) and transported into the cytoplasm by inner membrane ABC transporters (e.g., *Y. pestis* HmuU and HmuV or *Y. enterocolitica* HemU and HemV), powered by ATP hydrolysis (Wyckoff et al., 1998; Ho et al., 2007; Chu and Vogel, 2011). In the cytoplasm, HmuS or HemS proteins transfer heme to enzymes that either utilize heme or break heme down to release iron from heme (e.g., heme oxygenases) (Lansky et al., 2006; Schneider and Paoli, 2005; Schneider et al., 2006). It is worth noting that although the genes encoded on these operons and corresponding to the respective names participate in the process related to heme uptake, they are not necessarily homologous in the assigned function.

The main and best-characterized heme acquisition mechanism in *P. gingivalis* is the Hmu system, which is, on one side, different from typical heme uptake systems of Gram-negative bacteria and, on the other side, characteristic of the Bacteroidota phylum (Olczak et al., 2024; Śmiga and Olczak, 2025a,b). In *P. gingivalis*, the Hmu system consists of six proteins: well-characterized HmuY and HmuR, and four uncharacterized proteins (HmuS, HmuT, HmuU, and HmuV), all encoded on the *hmu* operon (Lewis et al., 2006; Olczak et al., 2008, 2024). HmuY is a unique hemophore-like heme-binding protein that captures heme from host hemoproteins and delivers it from the external environment to HmuR. HmuR is a typical TDR, enabling heme transport across the outer membrane. The third gene encodes HmuS, a protein homologous to CobN chelatases. HmuT is a membrane protein and a putative permease, and HmuU is homologous to the proton channel ExbB. The sixth gene of the *hmu* operon encodes an HmuV protein with no homology to any known protein family.

To release iron, heme is degraded aerobically by canonical heme oxygenases, examples being HemO from Gram-negative *Neisseria meningitidis* (Wilks and Heinzl, 2014; Wilks and Ikeda-Saito, 2014; Lyles and Eichenbaum, 2018; Richard et al., 2019). In this process, heme is converted into biliverdin and carbon monoxide, with ferric iron release and its conversion to ferrous iron (Matsui et al., 2010). Another group of enzymes comprises heme-binding, non-canonical heme-degrading proteins, including HemS from *Y. enterocolitica*, HmuS from *Yersinia pseudotuberculosis*, and ChuS from pathogenic enterohemorrhagic *Escherichia coli* O157:H7 (Onzuka et al., 2017; Lyles and Eichenbaum, 2018; Mathew et al., 2019; Keith et al., 2024). These proteins function as heme-degrading and iron-releasing enzymes under aerobic iron-deplete conditions but also participate in intracellular heme shuttling or protecting from oxidative stress as heme chaperones under anaerobic iron-replete conditions. As an example is *E. coli* K-12 EfeB (Létoffé et al., 2009). ChuW, an S-adenosylmethionine methyltransferase of *E. coli* O157:H7 or *Enterococcus faecalis*, degrades heme in the absence of oxygen using alternate electron donors (e.g., flavodoxin) and produces anaerobilin instead of biliverdin (LaMattina et al., 2016; LaMattina et al., 2017; Mathew et al., 2019; Brunson et al., 2025). Anaerobilin is then shuttled by ChuX from ChuW to ChuY for further breakdown. Recently, it has been shown that HemS from *Y. pestis* and HmuS from *Y. enterocolitica* degrade heme under anaerobic conditions using a NADH-dependent hydride transfer mechanism (Keith et al., 2024).

Heme is the main source of iron for *P. gingivalis*, but the exact mechanism of iron extraction from heme in this bacterium is not known. Among proteins potentially responsible for iron release from heme is the *P. gingivalis* HmuS (HmuS^Pg^), which is similar to its close homolog BtuS2 from *B. fragilis* (HmuS^Bf^) (Rocha et al., 2019) and *B. thetaiotaomicron* HmuS^Bt^ (Nath et al., 2025). This study aimed to characterize the *P. gingivalis* HmuS^Pg^ protein and compare its properties with the *B. fragilis* HmuS^Bf^ protein.

## 2 Materials and methods

### 2.1 Bacterial strains and growth conditions

*P. gingivalis* wild-type (W83) and modified strains (Table 1) were grown for 4 days at 37°C under anaerobic conditions (80% N_2_, 10% CO_2_, 10% H_2_; Whitley A35 anaerobic workstation; Bingley, United Kingdom) on blood agar plates (ABA) composed of Schaedler broth, supplemented with 5% sheep blood, hemin, L-cysteine and menadione (BioMaxima, Lublin, Poland). Bacterial colonies were inoculated into basal medium (BM) composed of 3% trypticase soy broth (Becton Dickinson, Sparks, MD, United States), 0.5% yeast extract (BioMaxima), 0.05 mg/L menadione (Fluka, Munich, Germany), and 0.05% L-cysteine (Sigma-Aldrich, St. Louis, MO, United States). The BM medium was supplemented with 7.7 μM hemin chloride (Hm medium) (Pol-Aura, Morag, Poland) to grow bacteria under heme-replete conditions. To grow bacteria with other heme sources, BM medium was supplemented with 2 μM hemoglobin (Hb medium) (Pol-Aura, Poland) or 7.7 μM human serum albumin (HSA) (Sigma-Aldrich) with 7.7 μM heme (HSA-Hm medium). To grow bacteria under iron and heme-depleted conditions, a heme source was not added, and the BM medium was supplemented with an iron chelator, 160 μM 2,2-dipyridyl (DIP medium) (Sigma-Aldrich). The mutant, control, and complemented strains were cultured in the presence of respective antibiotics (Table 1).

**TABLE 1:** List of *Porphyromonas gingivalis* strains examined in this study.

Strain name	Genotype	Antibiotic resistance (antibiotic concentration)	Description	Reference
W83	Wild-type	None	Wild-type strain	Laboratory collection
Δ*hmuS^Pg^*	*hmuS*^Δ^Tet^r^	Tetracycline (1 μg/mL)	Mutant strain with the *hmuS^Pg^* gene replaced by the *tetQ* antibiotic resistance gene without its native promoter (*tetQ* expression is under the native *hmu* operon promoter)	This study
WT + HmuS^Pg^	wild-type + pTIO-HmuS^Pg^	Erythromycin (3 μg/mL)	Control strain constructed in the wild-type W83 background, harboring the pTIO-HmuS^Pg^ plasmid, expressing both native HmuS^Pg^ and HmuS^Pg^-3 × HA proteins	This study
Δ*hmuS^Pg^* + HmuS^Pg^	*hmuS*^Δ^Tet^r^ + pTIO-HmuS^Pg^	Tetracycline (1 μg/mL), erythromycin (3 μg/mL)	Complemented strain constructed in the Δ*hmuS^Pg^* mutant strain, harboring the pTIO-HmuS^Pg^ plasmid, expressing HmuS^Pg^-3 × HA protein	This study

To monitor bacterial growth, bacteria were cultured in 96-well polystyrene flat-bottom plates (Sarstedt, Nümbrecht, Germany) in a liquid medium (200 μL per well), at a starting optical density of 600 nm (OD_600_) equal to 0.2. The growth was monitored over time using a Stratus plate reader (Cerillo, Charlottesville, VA, United States). The plate reader was placed inside an anaerobic chamber (80% N_2_, 10% CO_2_, 10% H_2_; 37°C; Whitley A35 anaerobic workstation) during the measurements.

*Escherichia coli* DH10B and BL21-CodonPlus (DE3)-RIL strains (Agilent Technologies, Santa Clara, CA, United States) were grown in Terrific Broth medium (TB; BioShop, Burlington, Ontario, Canada) under standard aerobic conditions.

### 2.2 Construction of the *hmuS^Pg^* deletion mutant strain

To generate the *hmuS^Pg^* deletion mutant strain (Δ*hmuS^Pg^*), a linear DNA construct composed of sequences flanking the target gene with an internally cloned antibiotic resistance cassette was prepared. The entire *hmuS^Pg^* gene (gene locus ID: PG1553/PG_RS06850) was replaced in the wild-type W83 strain with the tetracycline resistance gene (*tetQ*) from *Bacteroides thetaiotaomicron* (GenBank ID: X58717), lacking a native promoter and inserted in the same orientation as the *hmu* operon genes (Supplementary Figure S1A). The *tetQ* gene was amplified by PCR using the pTIO-tetQ plasmid (Śmiga et al., 2019) as a template. Flanking regions of the *hmuS^Pg^* gene were amplified by PCR using *P. gingivalis* W83 genomic DNA as a template. The obtained fragments were assembled using the NEBuilder HiFi DNA Assembly Kit (New England Biolabs, Ipswich, MA, United States). Subsequently, the linear construct was amplified by PCR and introduced into *P. gingivalis* wild-type W83 strain by electroporation (Simpson et al., 2000). All plasmids and primers are listed in Supplementary Tables S1, S2, respectively. All plasmids were propagated in the *E. coli* DH10B strain (Agilent Technologies). The mutant strain was selected on ABA plates supplemented with 1 μg/mL tetracycline. Homologous recombination between the linear construct and chromosomal DNA was verified by PCR (Supplementary Figure S1B) and DNA sequencing (Microsynth Seqlab GmbH, Gottingen, Germany).

### 2.3 Construction of the control and complemented strains

The complementation of the *hmuS^Pg^* gene deletion was carried out using the pTIO-HmuS^Pg^ plasmid, containing the *hmu* promoter, the gene encoding the full-length HmuS^Pg^ protein with a C-terminal 3 × HA tag, and the fragment of the downstream region of the *hmu* operon (Supplementary Figure S1C). The *hmu* promoter and most of the *hmuS^Pg^* gene were amplified by PCR using *P. gingivalis* W83 genomic DNA as a template. The fragment containing the 3’ end of the *hmuS^Pg^* gene, the sequence encoding the 3 × HA tag, and the fragment of the *hmu* operon downstream region were synthesized (GenScript, Rijswijk, Netherlands) (Supplementary Table S2) and cloned into the pUC57 plasmid (GenScript). After PCR amplification, this fragment, together with the *hmu* promoter and the majority of the *hmuS^Pg^* gene, was assembled with the pTIO-1 plasmid (Tagawa et al., 2014) (the latter digested with the *Xho*I and *Bam*HI restriction enzymes; New England Biolabs), using the NEBuilder HiFi DNA Assembly Kit (New England Biolabs), resulting in the pTIO-HmuS^Pg^ plasmid. All plasmids and primers are listed in Supplementary Tables S1, S2, respectively. The accuracy of the obtained construct was verified by PCR and DNA sequencing (Microsynth). pTIO-HmuS^Pg^ plasmid was used for electroporation of *P. gingivalis* wild-type W83 and Δ*hmuS^Pg^* mutant strains to get control (WT + HmuS^Pg^) and complemented (Δ*hmuS^Pg^* + HmuS^Pg^) strains. After electroporation, colonies were selected on ABA plates supplemented with appropriate antibiotics (Table 1).

### 2.4 Determination of HmuS^Pg^ localization and topology

To localize the HmuS^Pg^ in *P. gingivalis*, wild-type (WT), control (WT + HmuS^Pg^), and complemented (Δ*hmuS^Pg^* + HmuS^Pg^) strains were used. Bacteria were grown in Hm medium or DIP medium for 24 h in the presence of respective antibiotics (Table 1).

To localize HmuS^Pg^ in the *E. coli* model, a pTriEx-HmuS^Pg^ plasmid was constructed, containing the DNA sequence encoding full-length HmuS^Pg^ protein with a C-terminal 3 × HA-8 × His tag (Supplementary Figure S1D). The DNA fragment encoding HmuS^Pg^-3 × HA was amplified by PCR using the pTIO-HmuS^Pg^ plasmid as a template and cloned into the pTriEx-4 plasmid (Sigma-Aldrich) (the latter digested with *Nco*I and *Xho*I restriction enzymes; New England Biolabs), using the HiFi DNA Assembly Kit (New England Biolabs). The accuracy of the obtained construct was verified by PCR and DNA sequencing (Microsynth). The plasmid was used to transform the *E. coli* BL21-CodonPlus (DE3)-RIL strain (Agilent Technologies). Bacteria were grown at 37°C with shaking (220 rpm) in TB medium (BioShop) with the addition of 35 μg/mL chloramphenicol (Carl Roth) and 100 μg/mL carbenicillin (A&A Biotechnology, Gdansk, Poland) (37°C, 220 rpm) until OD_600_ ∼0.8 was reached. Protein expression was induced by 0.3 mM IPTG (Carl Roth), and the culture was incubated for ∼16 h at 16°C with shaking (220 rpm).

To localize the HmuS^Pg^, bacterial cells were fractionated using osmotic shock and differential centrifugation (Malherbe et al., 2019). For this purpose, the bacterial cultures were centrifuged (4,000 × *g*, 20 min, 4°C) and resuspended in 30 mM Tris-HCl buffer, pH 8.0, supplemented with 20% sucrose and 1 mM EDTA. Then, the samples were incubated for 10 min at 4°C and centrifuged (8,000 × *g*, 20 min, 4°C), resuspended in ice-cold 5 mM MgSO_4_, and incubated for 20 min at 4°C with gentle stirring. After centrifugation (8,000 × *g*, 20 min, 4°C), the supernatant containing the outer membrane (OM) and periplasmic fractions (P) was collected, while the pellet was suspended in 20 mM sodium phosphate buffer, pH 7.4, containing 140 mM NaCl (PBS), supplemented with 0.05% N-dodecyl-β-D-maltoside (Sigma-Aldrich). The suspension was lysed by sonication and centrifuged (20,000 × *g*, 20 min, 4°C). The supernatant contained the cytoplasmic (C) and inner membrane (IM) fractions. Both supernatants were further subjected to ultracentrifugation (150,000 × *g*, 16 h, 4°C). The resulting pellets, containing inner or outer membrane fraction, were resuspended in 100 mM sodium phosphate buffer, pH 7.4, containing 10% glycerol.

### 2.5 Overexpression and purification of recombinant proteins

The *hmuS^Pg^* gene, lacking the sequence encoding 39 N-terminal amino acid residues of the HmuS^Pg^ protein (40–1,469), was amplified by PCR using *P. gingivalis* W83 genomic DNA as a template and primers listed in Supplementary Table S2. To produce a recombinant protein with an N-terminal 8 × His and maltose-binding protein (His-MBP) (Supplementary Figure S2A), DNA fragments amplified by PCR were cloned into the pMAL-c5xHis plasmid (Śmiga et al., 2019) between the *Bam*HI and *Xmn*I restriction sites. Plasmid used to overexpress and purify homologous protein from *Bacteroides fragilis* NCTC 9343 (ATCC 25285) (HmuS^Bf^), lacking 27 N-terminal amino acid residues (28–1,439) and possessing an N-terminal His-MBP tag, was prepared analogously to the plasmid expressing HmuS^Pg^ (Supplementary Figure S2A), using *B. fragilis* genomic DNA as a template. As a control, His-MBP was overexpressed using the pMAL-c5xH plasmid (Śmiga et al., 2019). All plasmids and primers are listed in Supplementary Tables S1, S2, respectively. PCR and DNA sequencing (Microsynth) were carried out to verify the correctness of the constructs.

Expression plasmids were transformed into the *E. coli* BL21-CodonPlus (DE3)-RIL strain (Agilent Technologies). Bacteria were grown at 37°C with shaking (220 rpm) in TB medium (BioShop) with the addition of 35 μg/mL chloramphenicol and 100 μg/mL carbenicillin (37°C, 220 rpm) until OD_600_ ∼0.8 was reached. Protein overexpression was induced by 0.3 mM IPTG, and bacterial cultures were incubated for about 16 h at 16°C with shaking (220 rpm). Then, bacteria were centrifuged (4 000 × *g*, 20 min, 4°C) and the pellet was kept at −20°C until used.

To purify proteins, bacteria were suspended in 25 mM Tris-HCl buffer, pH 7.4, containing 300 mM NaCl, lysed with sonication (Sonopuls HD 4100; Bandelin, Berlin, Germany), and centrifuged (20,000 × *g*, 20 min, 4°C).

His-MBP-tagged recombinant protein and His-MBP protein were purified from the soluble bacterial fraction using TALON Superflow resin with immobilized cobalt (Sigma-Aldrich). After removing the unbound fraction, the resin was washed with 25 mM Tris-HCl buffer, pH 7.4, containing 300 mM NaCl and 5 mM imidazole, followed by 25 mM Tris-HCl buffer, pH 7.4, containing 1 M NaCl and 5 mM imidazole. His-MBP-tagged protein was eluted with 25 mM Tris-HCl buffer, pH 7.4, containing 300 mM NaCl and 200 mM imidazole. To re-purify the proteins initially purified by cobalt-affinity chromatography, the MBP-tagged recombinant protein was purified using amylose resin (New England Biolabs) according to the manufacturer’s instructions.

Purified proteins were concentrated using Amicon^®^ Ultra Centrifugal Filter (100 kDa MWCO; Millipore, Billerica, MA, United States) and stored in PBS, supplemented with 10% glycerol at −20°C until used.

N-terminally (His-MBP-HmuS^Pg^_C protein) and C-terminally (His-MBP-HmuS^Pg^_N protein) truncated HmuS variants (Supplementary Tables S1, S2) were overexpressed with His-MBP tag at their N-terminus, and purified as described for the His-MBP-HmuS^Pg^ protein.

### 2.6 Sodium dodecyl sulfate-polyacrylamide gel electrophoresis (SDS-PAGE), Western blotting, and dot blotting

Protein samples, bacterial cell lysates, or whole bacterial culture samples were prepared and analyzed using standard SDS-PAGE protocols. Samples containing bacterial cell lysates or whole bacterial cultures were prepared with a protease inhibitor cocktail (Bimake, Houston, TX, USA) and standardized to an OD_600_ or protein amount determined with Roti Nanoquant (Carl Roth), as previously reported (Śmiga et al., 2015, 2019). Proteins were separated using SDS-PAGE and either stained with Coomassie Brilliant Blue G-250 (CBB) or transferred onto nitrocellulose membranes (Millipore) using the Trans-Blot Turbo Transfer System (Bio-Rad Laboratories, Hercules, CA, United States). The efficiency of protein transfer was visualized on nitrocellulose membranes by staining proteins with Ponceau S (Sigma-Aldrich). To detect respective proteins, rabbit anti-HmuS^Pg^ (1:500) (antibodies were raised against purified recombinant His-MBP-HmuS^Pg^_N or His-MBP-HmuS^Pg^_C protein; ProteoGenix, Schiligheim, France), anti-IhtB protein (1:10,000; ProteoGenix), or mouse anti-HA (1:10 000; Sigma-Aldrich) antibodies were used. Subsequently, goat horseradish peroxidase (HRP)-conjugated anti-rabbit IgG (1:10,000; Sigma-Aldrich) or goat HRP-conjugated anti-mouse IgG (1:10,000; Proteintech, Rosemont, United States) antibodies were used. For dot blotting, the appropriate protein samples or bacterial cell lysates were applied onto nitrocellulose membranes and treated similarly to samples analyzed by Western blotting. The visualization of complexes was carried out using chemiluminescence staining (PerkinElmer, Waltham, MA, United States, or Thermo Fisher Scientific, Waltham, MA, United States) and a ChemiDoc imaging system (Bio-Rad Laboratories). Densitometric analysis (relative quantification) of protein levels on Western blots was performed using Image Lab 6.0.1 software (ChemiDoc, Bio-Rad). Results obtained for the mutant strain are shown in reference to those of the wild-type strain, with the latter set as 1.0.

### 2.7 Gene expression analysis using reverse transcriptase-quantitative polymerase chain reaction (RT-qPCR) and RNA sequencing (RNA-seq)

RNA was isolated from 1 mL of bacterial cultures grown in Hm medium and collected after 6 and 24 h, using a Total RNA Mini Kit (A&A Biotechnology, Gdańsk, Poland). Samples were treated with DNase and purified with the Clean-up RNA concentrator Kit (A&A Biotechnology).

For qPCR analysis, cDNA was synthesized using a LunaScript RT SuperMix Kit (New England Biolabs). qPCR was performed using the SensiFAST SYBR no-ROX Kit (Bioline, London, United Kingdom) and LightCycler 96 (Roche, Basel, Switzerland). The PCR program consisted of initial denaturation at 95°C for 120 s, and 40 cycles of denaturation at 95°C for 5 s, primer annealing at 60°C for 10 s, and extension at 72°C for 15 s. To assess the quality of PCR products, the melting curves were generated and analyzed. Changes in gene expression (fold change, FC) were calculated for three independent biological replicates using LightCycler 96 software (Roche) and *P. gingivalis 16S rRNA* (gene ID: PG_RS00460) as a reference gene. All primers are listed in Supplementary Table S2.

mRNA sequencing (RNA-seq) and data analysis were performed by Novogene (Cambridge, United Kingdom), according to the company’s standard protocols, using RNA samples isolated from bacteria grown in Hm medium for 24 h. Changes in gene expression (Fold change, FC) were calculated for three independent biological replicates. FC change was considered statistically significant when FC > 1.5 or FC < −1.5 was reached, and the *p*-value was lower than 0.05 (Supplementary Table S3).

### 2.8 Preparation of heme solution and analysis of heme binding

Heme (hemin chloride; Pol-Aura, Morag, Poland) was prepared in 0.1 M NaOH and its concentration was determined using the empirical extinction coefficient ε_385_ = 58.5 mM^–1^ cm^–1^ (Dawson et al., 1986). The formation of protein-heme complexes was examined in PBS. 5 μM protein solutions were mixed with heme at a 1:1 molar ratio. After 5-minute incubation at room temperature, the UV-visible spectra were recorded under oxidizing and reducing conditions. To maintain reducing conditions, sodium dithionite at a final 10 mM concentration was used under a mineral oil overlay. UV-visible spectra were recorded in the 250–700 nm range with a double-beam Jasco V-750 spectrophotometer (Jasco GmbH, Pfungstadt, Germany) using cuvettes with 10 mm path length. Difference spectra were prepared by subtracting the spectrum of heme alone from the protein-heme complex spectrum. All analyses were performed at least three times.

The abundance of heme binding to whole *P. gingivalis* cells was determined using bacteria cultured in Hm medium or DIP medium as described previously (Śmiga et al., 2023).

### 2.9 Heme and PPIX quantification in *P. gingivalis* cells

Bacteria were grown in Hm medium, collected after 6 and 24 h, centrifuged (4 000 × *g*, 20 min, 4°C), washed once with water, adjusted to OD_600_ equal to 1.25 and 2.5, respectively, sonicated, and lyophilized. The dry mass of lyophilized bacterial lysates was reconstituted in an extraction solution composed of acetonitrile (ACN)/12 M HCl/DMSO (41:9:50, v/v/v) to achieve a final concentration of 180 μg of lysate per 300 μL of solvent (da Silva et al., 2024). The reconstituted samples were then centrifuged (12,000 rpm, 10 min, 25°C), and supernatants were loaded onto a Hypersil GOLD column (4.6 mm × 250 mm, 5 μm particle size; Thermo Fisher Scientific) connected to the HPLC system (Prominence-i LC-2030C 3D Plus; Shimadzu, Columbia, MD, United States). The chromatography was carried out using a linear gradient with solution A [ultrapure water + 0.1% trifluoroacetic acid (TFA)] and solution B (ACN + 0.1% TFA) at a flow rate of 1 mL/min at 25°C. For absolute quantification, a standard curve was generated using hemin chloride or PPIX (da Silva et al., 2024). Experiments were conducted in four repetitions, each using three biological replicates.

### 2.10 Pigment extraction and targeted liquid chromatography-mass spectrometry (LC-MS) analysis

Aliquots of 20 μL of *P. gingivalis* cultures at an OD_600_ of 2.0 were plated on ABA plates and cultured anaerobically for 6 days at 37°C. The pigment was extracted from ∼ 20 mg of bacterial colonies using 100 μL of 100% methanol and glass tubes, and kept in the dark. Samples were pipetted for 1 min and then centrifuged (4,000 × *g*, 10 min, 4°C). The extracted pigment was examined using LC-MS performed on an M-Class Acquity UPLC system coupled to a Synapt XS HDMS equipped with an ESI ion source interface (Waters, Milford, MA, United States). Mobile phase A consisted of 0.1% formic acid (FA) and 0.05% TFA in water, while mobile phase B consisted of 0.1% FA + 0.05% TFA in ACN (all solvents and additives were LC-MS grade; WITKO, Łódź, Poland). 2 μL of undiluted pigment sample was injected, desalted on-system, and a 25-min 10–99% B linear gradient at a flow rate of 50 μL/min was applied for sample separation on an Acquity BEH C18 130 Å, 1.7 μm, 1 mm × 100 mm column (Waters), which was kept at 60°C. Three independent biological extract replicates were analyzed for each strain (*n* = 3). Full scan MS data was collected at 1 s/scan through a 150–1,000 m/z range in ESI + with Analyzer mode set to Resolution. A Glu-1-Fibrinopeptide B solution (Waters) was acquired in the reference function, and the correction was applied in-acquisition.

Qualitative confirmation of compounds of interest was carried out in MassLynx v4.1 (Waters), while their relative quantitation was performed using Skyline v25.1.0.237.^1^ Heme and PPIX retention times and adduction mode were assessed by running commercial standards (hemin, cat. no. PA-03-9985-E, Pol-Aura, Zabrze, Poland; PPIX, cat. no. P8293, Sigma-Aldrich). Qualitative confirmation also included an isotopic model comparison. For the ion chromatogram extraction (XIC) in Skyline, a 0.05 m/z tolerance window was applied (albeit all peaks were matched at < ± 3 ppm), and peak intensities were total ion current (TIC) normalized between samples.

### 2.11 Determination of gingipain activity

Gingipain activity was determined using lysine-(N-(p-tosyl)-Gly-Pro-Lys 4-nitroanilide acetate salt; Sigma-Aldrich) and arginine-specific (Nα-benzoyl-DL-arginine p-nitroanilide hydrochloride; Sigma-Aldrich) substrates for Kgp and Rgp gingipains, respectively (Pomowski et al., 2017; Śmiga et al., 2024a), with minor modifications. Briefly, 150 μL of the reaction buffer (20 mM Tris-HCl buffer, pH 7.5, supplemented with 150 mM NaCl, 0.05% Tween 20, 5 mM CaCl_2_, and 10 mM L-cysteine hydrochloride, neutralized with NaOH) was mixed with 10 μL of *P. gingivalis* cultures and the appropriate substrates. Samples were incubated for 2 h at 37°C, and the reaction was monitored by measuring the increase in absorbance at 405 nm (A_405_) over time using a GloMax Discover plate reader (Promega). The enzymatic activity was calculated from the linear part of the A_405_ curve versus time and is shown as the increase in the product absorbance determined at 405 nm (mOD) in one min by 1 μL of the *P. gingivalis* culture of OD_600_ = 1.0 [mOD/min/μL].

### 2.12 Analysis of potential reverse ferrochelatase activity

The potential enzymatic activity was measured by monitoring the decrease in heme content through changes in the UV-visible absorbance spectrum (350–700 nm) over time. Enzymatic activity was measured in the samples containing 10 μM His-MBP-HmuS^Pg^ or 10 μM His-MBP-HmuS^Bf^ in 100 mM phosphate buffer, pH 8.0, supplemented with 40 μM NADH, 10 mM sodium dithionite, and 20 μM heme. The reaction was carried out in the absence or presence of purified *P. gingivalis* or *E. coli* inner membrane fraction obtained as described for the determination of HmuS^Pg^ localization. The amount of the added inner membrane corresponded to bacterial cultures at an OD_600_ equal to 0.5. Purified His-MBP protein was used as a control to show the reaction specificity for the HmuS proteins.

### 2.13 Determination of iron content

Intracellular free iron content was determined using the modified ferrozine-based method in *P. gingivalis* grown in Hm medium or DIP medium as described previously (Śmiga et al., 2023).

Total iron content was analyzed by atomic absorption spectroscopy (Agilent 200 series; Agilent) with flame generated from acetylene and compressed air. The samples (100 μL) were diluted in deionized water (1.5 mL), mixed with 190 μL of 20% nitric acid, heated for 5 min at 95°C, and loaded onto an atomic absorption spectrometer (Meslé et al., 2023). To quantify iron, a standard curve correlating absorbance and iron concentration (ppm) was generated using AA standards (Ricca; 100 ppm; CAS AFE1KH-100) diluted from 0.1 to 2 ppm in deionized water (Meslé et al., 2023).

### 2.14 Biofilm formation on an abiotic surface

Biofilm formation was analyzed as reported previously (Śmiga et al., 2024a) with minor modifications. Briefly, *P. gingivalis* was cultured for 2 passages in Hm medium. Then, fresh Hm medium or DIP medium was inoculated with bacterial cultures at an OD_600_ equal to 0.2. 1.2 mL of bacterial culture was added per well of a 24-well, polystyrene, flat-bottom plate (Corning, New York, United States). Bacteria were grown anaerobically for 48 h at 37°C. Unattached bacteria were removed by washing wells three times with PBS. The biofilm-forming bacteria were stained with 600 μL of 1% crystal violet (Carl Roth) for 30 min and then washed 5 times with PBS. Biofilms were de-stained with 200 μL of 96% ethanol by pipetting, and sample absorbance was measured at 570 nm (A_570_) using a GloMax Discover plate reader (Promega). Biofilm formation was normalized to the OD_600_ of bacterial cultures simultaneously grown for 48 h.

### 2.15 Theoretical and statistical analyses

Protein amino acid sequences were obtained from the Protein database.^2^ The search for the HmuS^Pg^ (protein sequence ID: AKV63660.1) homologs was performed using PSI-BLAST. The sequences were selected manually. Protein amino acid sequences were compared using Clustal Omega (the Multiple Sequence Alignment tool) (Madeira et al., 2024).

The phylogenetic tree was created using the Simple Phylogeny tool (Madeira et al., 2024) and visualized using iTOL (Letunic and Bork, 2024).

The protein topology was predicted with DeepTMHMM (Hallgren et al., 2022).

The theoretical structures of the HmuS^Pg^ and HmuS^Bf^ proteins, and their heme-bound variants, were modeled using the AlphaFold 3.0 server^3^ (Jumper et al., 2021; Varadi et al., 2022). Using the HmuS^Pg^ amino acid sequence, the protein structure was modeled both in the absence and in the presence of one or two heme ligands. Multiple structural predictions were generated, and those with the highest confidence scores were selected, showing consistent ligand positioning across independent predictions and geometrically realistic coordination distances between the heme iron and the predicted amino acid residues.

The structures were visualized with the UCSF ChimeraX program^4^ (Pettersen et al., 2004). The CobN cobaltochelatase from *Mycobacterium tuberculosis* (PDB ID: 7C6O) was used as a template for modeling the HmuS^Pg^ protein, while the magnesium chelatase ChlH from *Synechocystis* sp. (PDB ID: 4ZHJ) was used for the HmuS^Bf^ protein.

All statistical analyses were performed with GraphPad software (GraphPad Prism 8.0 Inc., San Diego, CA, United States) using the unpaired Student’s *t*-test or one-way analysis of variance (ANOVA) test with *post hoc* Tukey’s test. To assess the significance of differences in protein levels detected by Western blotting and in biofilm formation, a one-sample Student’s *t*-test was performed on three biological replicates, normalized to the mean values of the wild-type strain. The growth curves were analyzed using two-way ANOVA. All experiments were conducted at least three times with at least three biological replicates, and the collected data are shown as mean ± standard deviation (mean ± SD) or as mean ± standard error (mean ± SE).

## 3 Results

### 3.1 HmuS^Pg^ belongs to a chelatase family

**FIGURE 1:**
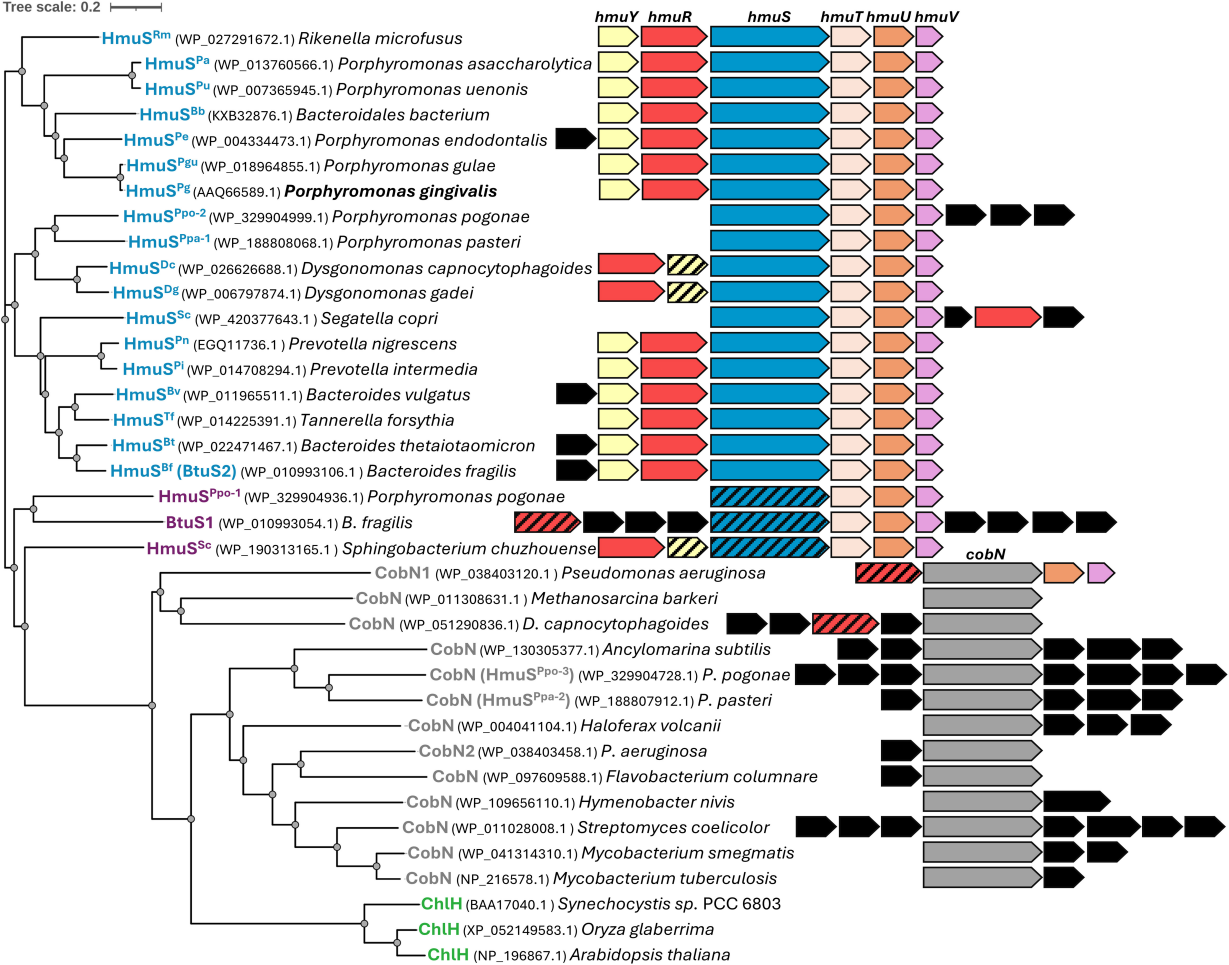
Phylogenetic analysis of selected HmuS, CobN, and ChlH chelatases. The phylogenetic tree was constructed based on amino acid sequences. The tree includes protein names (with their sequence IDs in brackets) alongside the corresponding bacterial species names. A schematic presentation of operons encoding the proteins is shown. Genes encoding proteins homologous to those encoded on the *Porphyromonas gingivalis hmu* operon are shown in yellow (*hmuY*), red (*hmuR*), blue (*hmuS*), beige (*hmuT*), orange (*hmuU*), or pink (*hmuV*). Genes encoding proteins that are not homologous to the Hmu proteins are shown in black. Genes homologous to those encoded on the *P. gingivalis hmu* operon but likely serving different functions are marked with black diagonal dashes. *cobN* genes are marked in gray. In plants and *Synechocystis* sp. (written in green), ChlH is encoded by a single gene.

HmuS proteins are conservatively preserved within members of the Bacteroidota phylum (Olczak et al., 2024; Śmiga and Olczak, 2025b), but their function is yet to be defined. They are a group of proteins composed of ∼1,450 amino acid residues of a molecular mass of ∼165 kDa. *P. gingivalis* HmuS^Pg^ (GenBank ID: AAQ66589) shows amino acid sequence similarity to CobN cobaltochelatase and the magnesium chelatase family proteins, and belongs to a chelatase family together with other HmuS homologs from the Bacteroidota phylum. It is closely related to the *B. fragilis* HmuS^Bf^ (BtuS2) and *B. thetaiotaomicron* HmuS^Bt^ proteins (Figure 1 and Supplementary Figure S3A; Rocha et al., 2019; Nath et al., 2025). HmuS^Pg^ exhibits 47.35% amino acid sequence identity to HmuS^Bf^ from *B. fragilis*; therefore, in our research model, we used HmuS^Bf^ as a reference protein, which has not been previously biochemically characterized. Our phylogenetic analyses showed that, when comparing amino acid sequences of HmuS proteins from the Bacteroidota phylum with representative CobN cobaltochelatases and ChlH magnesium chelatases, they form a separate protein group (Figure 1).

HmuS^Pg^ is encoded on the *hmu* operon, and most of the HmuS proteins in other species are encoded on *hmu* operons or *hmu*-like gene clusters (Figure 1). In contrast, CobN proteins are encoded on operons that do not encode proteins homologous to those of the Hmu system. Moreover, some of the analyzed Bacteroidota species encode two (e.g., *B. fragilis*, *Porphyromonas pasteri*) or even three (e.g., *Porphyromonas pogonae*) HmuS homologs ( Śmiga and Olczak, 2025b), which are classified into other protein clades, thereby suggesting their distinct functions.

**FIGURE 2:**
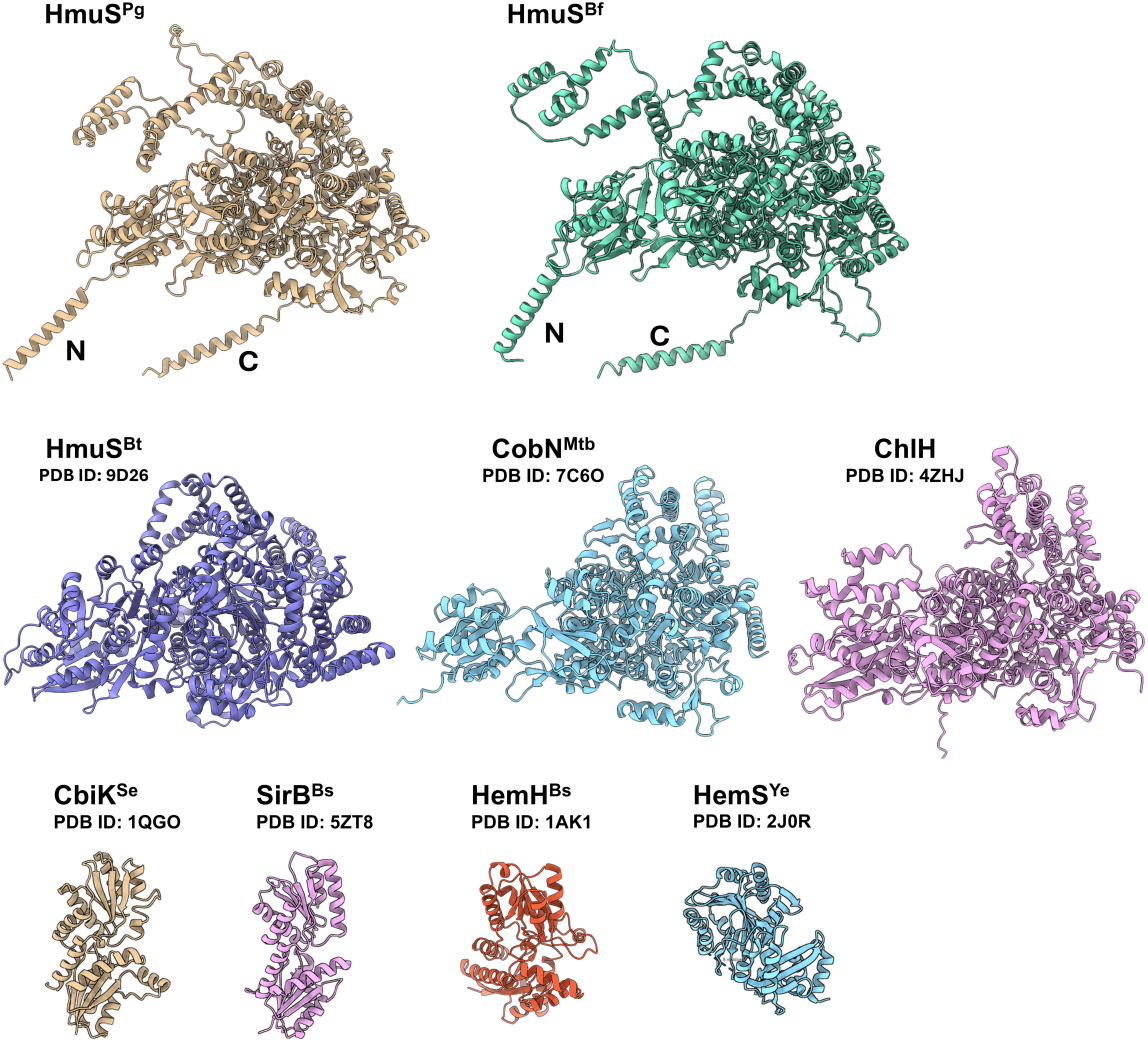
Three-dimensional structures of chosen chelatases or heme-degrading enzymes. Protein structures were obtained from the RCSB PDB homepage (https://www.rcsb.org) or modeled using AlphaFold (https://alphafold.com) and visualized with UCSF ChimeraX (see text footnote 4). HmuS^Pg^ from *Porphyromonas gingivalis*, HmuS^Bf^ from *Bacteroides fragilis*, HmuS^Bt^ from *Bacteroides thetaiotaomicron*, CobN^Mtb^ from *Mycobacterium tuberculosis*, ChlH from *Synechocystis* sp. PCC 6803, CbiK from *Salmonella enterica*, SirB^Bs^ from *Bacillus subtilis*, HemH^Bs^ from *B. subtilis*, HemS^Ye^ from *Yersinia enterocolitica*. N and C indicate the N- and C-terminus membrane fragment of the protein, respectively.

Theoretical analyses of the modeled three-dimensional protein structure showed that HmuS^Pg^ is highly similar to the *B. thetaiotaomicron* HmuS^Bt^ (47.20% amino acid sequence identity), whose structure was recently solved (PDB ID: 9D26) (Nath et al., 2025). It also shows similarity to the CobN from *Mycobacterium tuberculosis* (PDB ID: 7C6O) (Zhang et al., 2021) and ChlH from *Synechocystis* sp. PCC 6803 (PDB ID: 4ZHJ) (Chen et al., 2015) (Figure 2). No similarity to other classes of chelatases (e.g., cobaltochelatase CbiK, ferrochelatase HemH) or iron-releasing proteins (e.g., HmuS/HemS from *Yersinia* species) was found (Figure 2).

**FIGURE 3:**
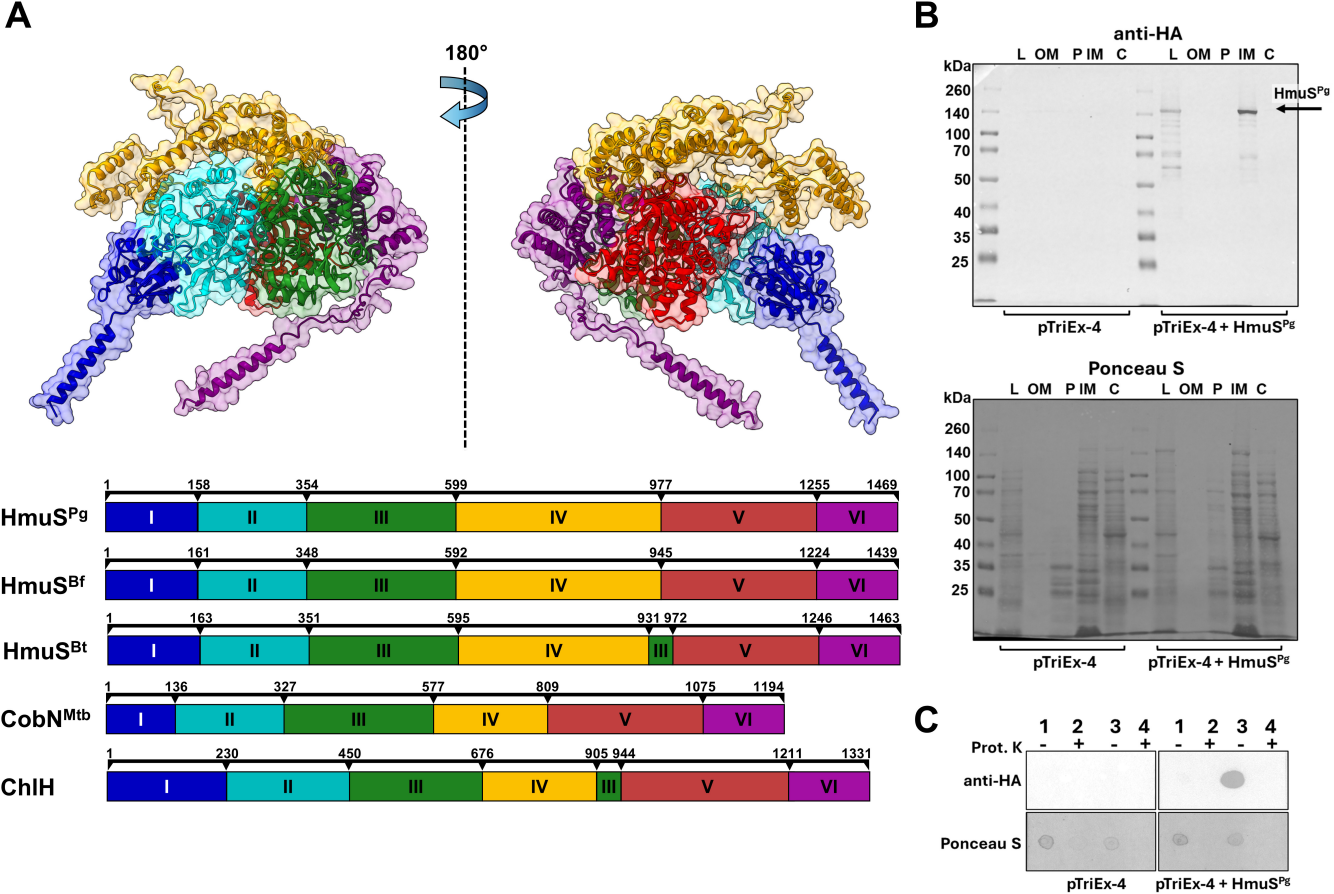
*Porphyromonas gingivalis* HmuS^Pg^ structure and localization. **(A)** The three-dimensional protein structure was predicted using AlphaFold. HmuS^Pg^ protein, similar to the *Bacteroides fragilis* HmuS^Bf^ protein, can be divided into six domains (I-IV), analogous to those of *Bacteroides thetaiotaomicron* HmuS^Bt^ (PDB ID: 9D26), *Mycobacterium tuberculosis* CobN^Mtb^ (PDB ID: 7C6O), and ChlH from *Synechocystis* sp. PCC 6803 (PDB ID: 4ZHJ). The scales above the schematic domain composition indicate the amino acid positions in the protein sequences. The HmuS^Pg^ localization was analyzed in the *Escherichia coli* model **(B,C)**. *E. coli* cells producing HmuS^Pg^ protein (pTriEx-HmuS^Pg^) and a control *E. coli* strain harboring the empty plasmid (pTriEx-4) were examined. **(B)** The recombinant HmuS^Pg^-HA-His (HmuS^Pg^) protein was detected in bacterial cell fractions using Western blotting with anti-HA antibodies. M, protein molecular marker; L, whole cell lysate; OM, outer membrane; P, periplasmic fraction; IM, inner membrane; C, cytosolic fraction. **(C)** Detection of HmuS^Pg^-HA-His in *E. coli* cell fractions using dot blotting and anti-HA antibodies. 1, cells without outer membrane; 2, cells without outer membrane after proteinase K (Prot. K) digestion; 3, cells without outer membrane after lysis; 4, cells without outer membrane after lysis and proteinase K digestion. Digestion with proteinase K was carried out to determine the topology of the HmuS^Pg^ protein.

Similar to CobN and ChlH proteins, the HmuS^Pg^ protein can be divided into six domains (I-VI), analogous to those found in the HmuS^Bf^ protein (Figure 3A). These findings were supported by comparative analysis performed using the HmuS^Bt^ protein structure (PDB ID: 9D26). The largest differences between HmuS and CobN or ChlH protein groups are found in domain IV, which is nearly twice as long and is rich in methionine residues in HmuS proteins (Figure 3A and Supplementary Figure S3A). Notably, variations are also observed among HmuS proteins themselves. Compared with HmuS^Bt^, HmuS^Pg^ and HmuS^Bf^ display distinct structural features, particularly in the helices that form surface-exposed loops, mainly within domain IV. In HmuS^Pg^, this region forms an extended loop that protrudes from the protein structure (Figures 2, 3A). This may result from actual differences in amino acid sequence (Supplementary Figure S3A) or protein structure (Figure 2), which may not entirely represent an accurate HmuS^Pg^ and HmuS^Bf^ structures through modeling (this study), or a disordered region not visible in the HmuS^Bt^ cryo-electron microscopy structure (Nath et al., 2025).

### 3.2 HmuS^Pg^ may function in the periplasmic space

Theoretical analyses showed that the cellular localization of HmuS proteins is different from that of CobN and ChlH. HmuS^Pg^ and its close homologs (HmuS^Bf^ and HmuS^Bt^) have a signal peptide at the N-terminus and transmembrane α-helices at the N- and C-termini (Figure 2 and Supplementary Figure S4), indicating that they are membrane-anchored proteins. In contrast, CobN and ChlH lack N- or C-terminal regions predicted to form membrane-spanning α-helices (Supplementary Figures S3A, S4A), indicating that they are soluble proteins localized in the cytoplasm, which is in accordance with their function in cobalamin and chlorophyll synthesis, respectively. Interestingly, although it was shown that *Synechocystis* sp. PCC 6803 ChlH can associate with membrane fractions in a Mg^2+^-dependent manner (Osanai et al., 2009), it is not an integral membrane protein, but rather its localization is a phenomenon occurring due to the protein’s interaction with membrane lipids or membrane proteins.

Attempts to localize the native HmuS^Pg^ in the *P. gingivalis* wild-type strain were unsuccessful due to the very low specificity and sensitivity of polyclonal antibodies raised against purified HmuS^Pg^ N- (40–858 amino acids) or C-terminal (899–1,469 amino acids) protein fragments (data not shown). Therefore, the complemented (Δ*hmuS^Pg^* + HmuS^Pg^) and the control (WT + HmuS^Pg^) strain were constructed, producing HmuS^Pg^-HA protein. *hmuS^Pg^* expression was detected in both the Δ*hmuS^Pg^* + HmuS^Pg^ and WT + HmuS^Pg^ strains grown in the Hm and DIP media. Relative to the wild-type strain, transcript levels in Hm medium increased 1.14 ± 0.33-fold in the Δ*hmuS^Pg^* + HmuS^Pg^ strain and 2.45 ± 1.57-fold in the WT + HmuS^Pg^ strain, whereas growth in DIP medium resulted in higher *hmuS^Pg^* expression, with fold changes of 5.65 ± 1.34 and 14.96 ± 11.97, respectively. Despite the increased mRNA levels, HmuS^Pg^-HA protein could not be detected using anti-HA antibodies, most likely due to low protein abundance or limited accessibility of the HA epitope (data not shown).

Therefore, we used the overexpressed HmuS^Pg^-HA-His protein variant to demonstrate HmuS^Pg^ localization in *E. coli*. We chose this model because in the previous study (Olczak et al., 2008), *P. gingivalis* HmuY and HmuR proteins were produced and localized correctly in the outer membrane of *E. coli*, and their function was confirmed by heme binding to the bacterial cell surface. As shown in Figure 3B, the protein may be localized in the *E. coli* inner membrane. The topology analysis of the C-terminus showed that the anchored protein may also be exposed to the periplasmic space (Figure 3C), thus confirming the theoretical analyses.

Although native HmuS^Pg^ could not be detected in *P. gingivalis*, several independent lines of evidence suggest a possible native localization and exposure of this protein. Therefore, the localization results obtained in *E. coli* should be interpreted as supporting evidence for the proposed topological model rather than as definitive confirmation of subcellular localization in *P. gingivalis*.

### 3.3 HmuS^Pg^ is a heme-binding protein engaged in heme metabolism

To characterize the HmuS^Pg^ protein, it was overexpressed using an *E. coli*-based system. As the protein was highly susceptible to degradation, to increase protein solubility and stability, we overexpressed and purified recombinant HmuS^Pg^ with His-MBP at the N-terminus (His-MBP-HmuS^Pg^; Supplementary Figure S2A). Despite this, purification procedures yielded non-homogeneous, enriched protein samples with a tendency to protein degradation (Supplementary Figure S2B). Attempts to remove the tag caused protein instability and precipitation. A similar result was obtained for the His-MBP-HmuS^Bf^ protein (Supplementary Figure S2B).

When His-MBP-HmuS^Pg^ and His-MBP-HmuS^Bf^ were purified from *E. coli* cell lysate and concentrated, they showed a color with different intensities, resulting in UV-visible spectra characteristic of protein-porphyrin complexes (Supplementary Figure S5). Theoretical analyses showed, in fact, two potential heme-binding sites in HmuS^Pg^ and other HmuS proteins (Supplementary Figure S3), including Met75 and His252 (site I) and His544 and His1217 (site II) in HmuS^Pg^.

**FIGURE 4:**
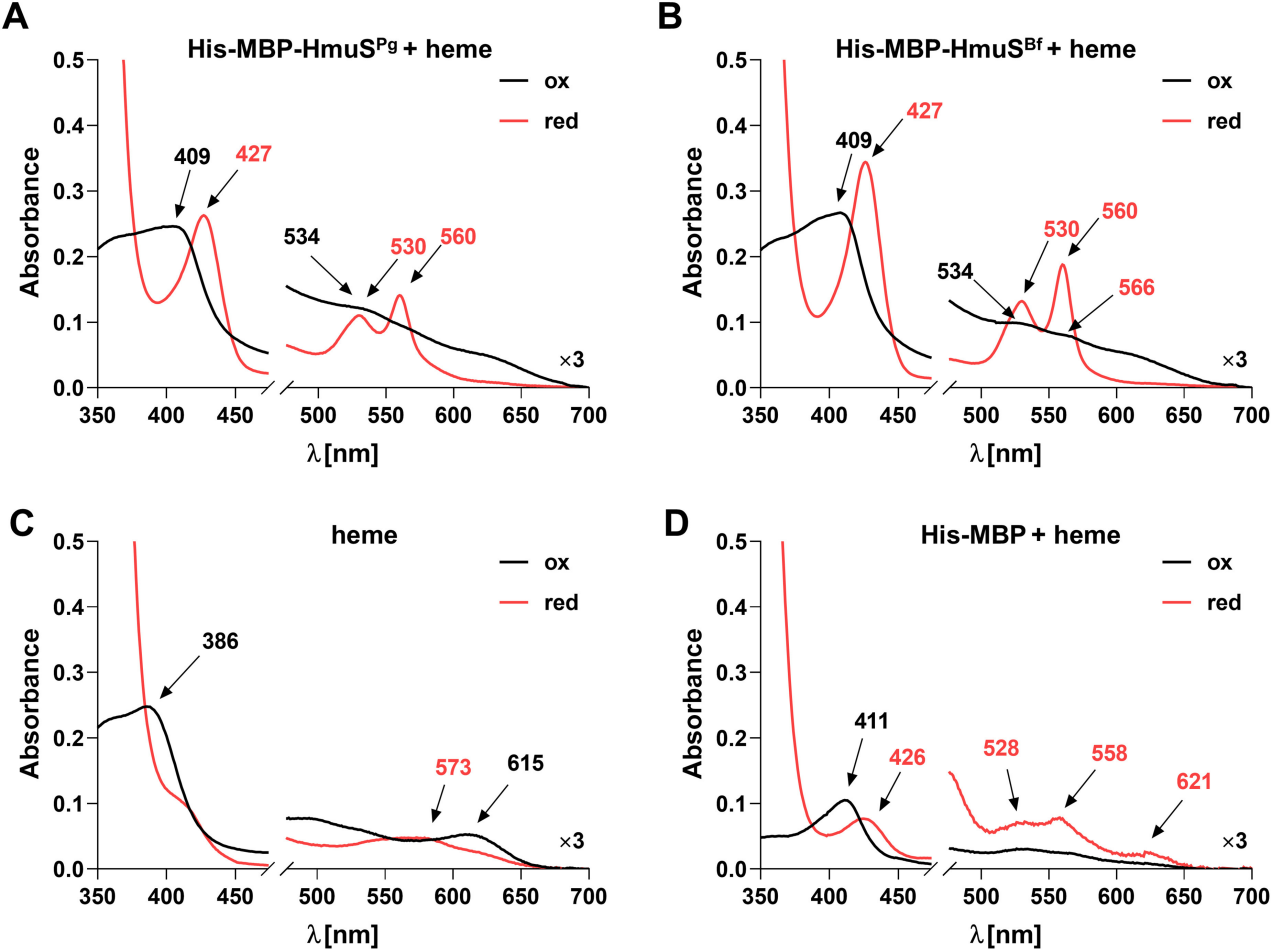
Heme binding to *Porphyromonas gingivalis* HmuS^Pg^ **(A)** and its homolog from *Bacteroides fragilis* (HmuS^Bf^) **(B)**. Heme binding was analyzed by adding 5 μM heme to 5 μM protein (1:1 molar ratio) and recording the UV-visible spectra. Protein-heme complexes were examined under oxidizing (ox) and reducing (red) conditions, the latter created by adding sodium dithionite to a final concentration of 10 mM and keeping the samples under mineral oil overlay. Heme alone **(C)** and heme mixed with His-MBP protein **(D)** were used as controls. The experiment was conducted three times, and one representative spectrum is shown.

When heme was added to the His-MBP-HmuS^Pg^ protein sample at a protein:heme 1:1 molar ratio, the maximum in the Soret region at 409 nm and a hardly visible maximum in the Q band region at 534 nm were observed under oxidizing conditions (Figure 4A). The reduction of the samples resulted in a red shift with the maximum in the Soret region at 427 nm and maxima in the Q band region at 530 and 560 nm (Figure 4A). The His-MBP-HmuS^Bf^ protein had not been previously biochemically characterized; therefore, its ability to bind heme was analyzed. The pattern of UV-visible spectra was similar (Figure 4B and Supplementary Figure S5) to that recorded for the His-MBP-HmuS^Pg^ protein (Figure 4B and Supplementary Figure S5), confirming heme binding. Analysis of heme alone Figure 4C confirmed heme interaction with proteins (Figures 4A,B).

Since there was a concern that His-MBP alone may influence heme binding, the protein was examined as described above. UV-visible spectroscopy showed that His-MBP can bind heme (especially under reducing conditions), but with a very low ability compared to His-MBP-HmuS^Pg^ and His-MBP-HmuS^Bf^ proteins (Figure 4D and Supplementary Figure S5).

**FIGURE 5:**
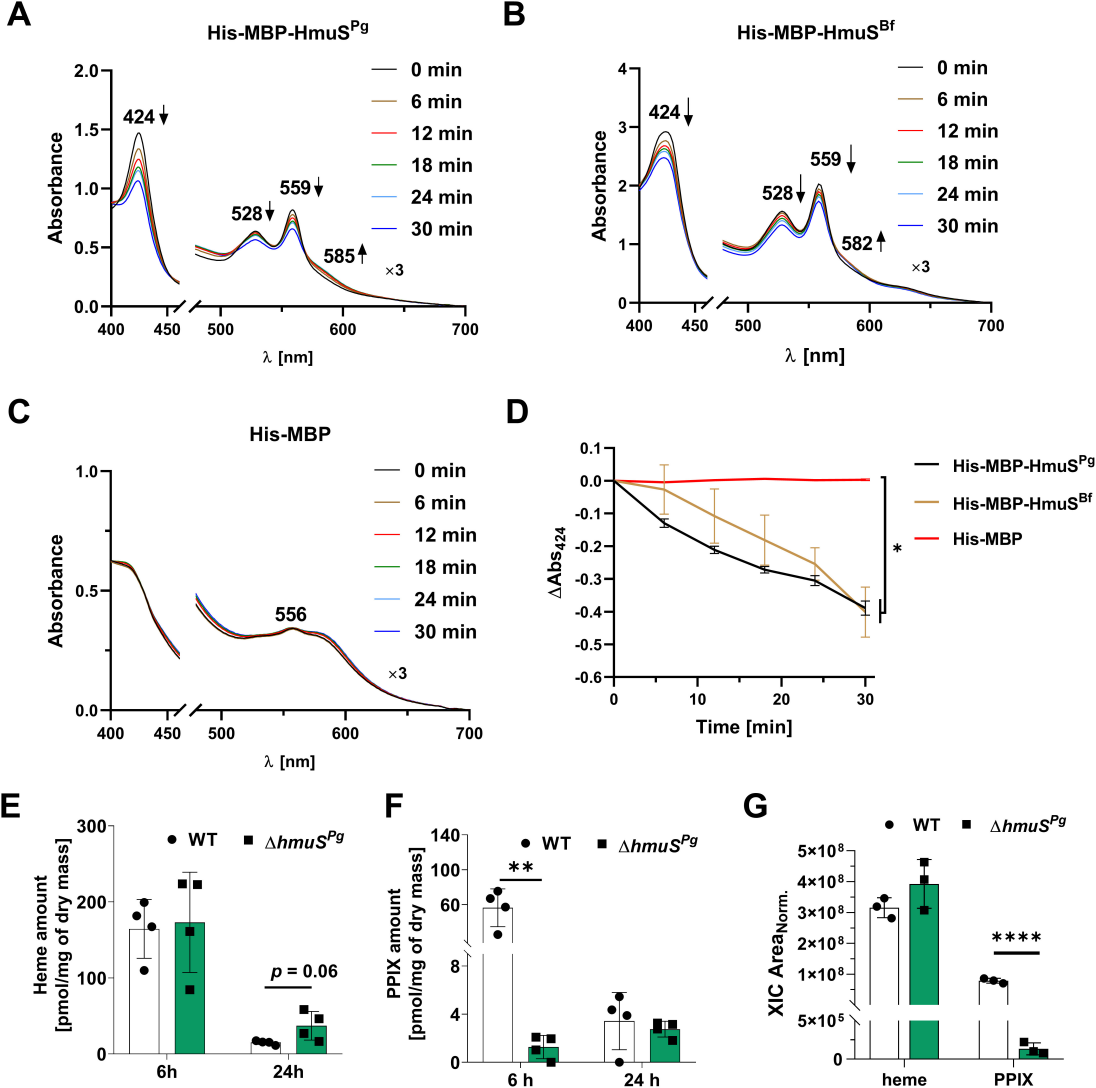
Analysis of heme metabolism. Visible spectra of *Porphyromonas gingivalis* His-MBP-HmuS^Pg^ **(A)**, *Bacteroides fragilis* His-MBP-HmuS^Bf^ **(B)**, and His-MBP **(C)**, incubated with heme, NADH, and inner membrane fraction, the latter isolated from the *P. gingivalis* wild-type strain, were analyzed over time. Controls confirming the specificity of the reaction are presented in Figure S6. The changes in the spectra are indicated with arrows. **(D)** A decrease in heme content was determined as the difference in the absorbance at 424 nm (ΔAbs_424_) at the initial time subtracted from the absorbance at a given time point. Heme **(E)** and PPIX **(F)** contents were determined in the *P. gingivalis* wild-type (WT) and mutant (Δ*hmuS^Pg^*) strains grown for 6 or 24 h under heme-rich conditions (Hm medium). Porphyrins extracted from whole bacterial cells were analyzed using normal-phase chromatography and standard curves. The results are shown as pmol of the respective porphyrin per mg of dry bacterial mass. **(G)** The effect of the Δ*hmuS^Pg^* deletion on the porphyrin content in the pigment deposited on *P. gingivalis* cells. The pigment was extracted from bacteria grown on blood agar plates using 100% methanol and analyzed by LC-MS (details are presented in Supplementary Figure S7). Bars represent the extracted ion chromatogram (XIC) peak areas of each compound. Peak areas were normalized to the total ion current (TIC). *p < 0.05; **p < 0.01; ****p < 0.0001.

To reveal the engagement of HmuS proteins in heme metabolism, the changes in the visible spectra of the HmuS-heme complexes over time were monitored (Figures 5A–D). They suggested the conversion of heme, catalyzed by the His-MBP-HmuS^Pg^ or His-MBP-HmuS^Bf^ proteins. In addition, the *in vitro* reaction required NADH, which is in agreement with the requirements of similar enzymes (Keith et al., 2024; Nath et al., 2025). NADH acts as a hydride donor and a reducing agent on ferric iron bound to porphyrins. *In vivo*, HmuS^Pg^ may require a membrane electron transfer chain (e.g., quinones, flavoproteins) to provide reducing equivalents to the periplasmic space.

Moreover, HmuS^Pg^ activity was stimulated by the inner membrane fraction isolated from *P. gingivalis* cells (Supplementary Figure S6). During the incubation, the absorbance of the Soret band at 424 nm and Q band at 528 and 559 nm decreased for both His-MBP-HmuS^Pg^ and His-MBP-HmuS^Bf^ proteins, suggesting a decrease in the heme amount (Figures 5A,B). This effect was not visible in control samples (Figures 5C,D and Supplementary Figure S6), confirming the specificity of the reaction. Analysis carried out with the *E. coli* inner membrane fraction yielded UV-visible spectra similar to those obtained using the *P. gingivalis* inner membrane fraction, but the reaction efficiency was much lower (Supplementary Figure S6F).

Subsequently, we analyzed whether *hmuS^Pg^* gene deletion would influence the heme and PPIX content in *P. gingivalis* cells. For this purpose, the heme and PPIX amounts were determined in the cell lysates derived from bacteria grown under heme-replete conditions (Hm medium). Although no statistically significant differences were determined in the amount of heme (Figure 5E), the content of PPIX was significantly lower in the Δ*hmuS^Pg^* mutant strain compared to the wild-type strain, especially at 6 h (Figure 5F). Moreover, the examination of the pigment composition revealed a significantly lower PPIX amount in the pigment deposited on the surface of the Δ*hmuS^Pg^* mutant strain (Figure 5G and Supplementary Figure S7). A decrease in the PPIX may indicate that the inactivation of the *hmuS^Pg^* gene resulted in a disturbance in iron removal from heme.

### 3.4 Inactivation of the *hmuS^Pg^* gene induces transcriptional remodeling of iron/heme transport systems and bacterial virulence potential

Heme availability influences the expression and production of HmuY and HmuR proteins (Olczak et al., 2008; Śmiga et al., 2023). In this study, we analyzed whether this is the case for the *hmuS^Pg^* gene under the conditions applied. *P. gingivalis* grown under heme-rich conditions (Hm medium) and then cultured in iron and heme-depleted conditions (DIP medium) for 6 or 24 h produced higher levels of the *hmuS^Pg^* mRNA (fold change 2.36 ± 0.5 or 13.59 ± 3.81, respectively). These findings showed that the *hmuS^Pg^* gene is preferentially expressed under low iron and heme conditions.

To verify the role of the HmuS protein in *P. gingivalis*, the phenotype of the Δ*hmuS^Pg^* strain was analyzed; however, surprisingly, there were no differences in the growth rate, despite using different media with diverse heme sources, including free heme, hemoglobin, albumin-heme complex, or blood (Supplementary Figures S8A,B). Moreover, the inactivation of the *hmuS^Pg^* gene has not been reflected in different heme adsorption by bacterial surface structures (Supplementary Figure S8C) or free and total iron contents (Supplementary Figures S8D,E), as compared to the wild-type strain.

**FIGURE 6:**
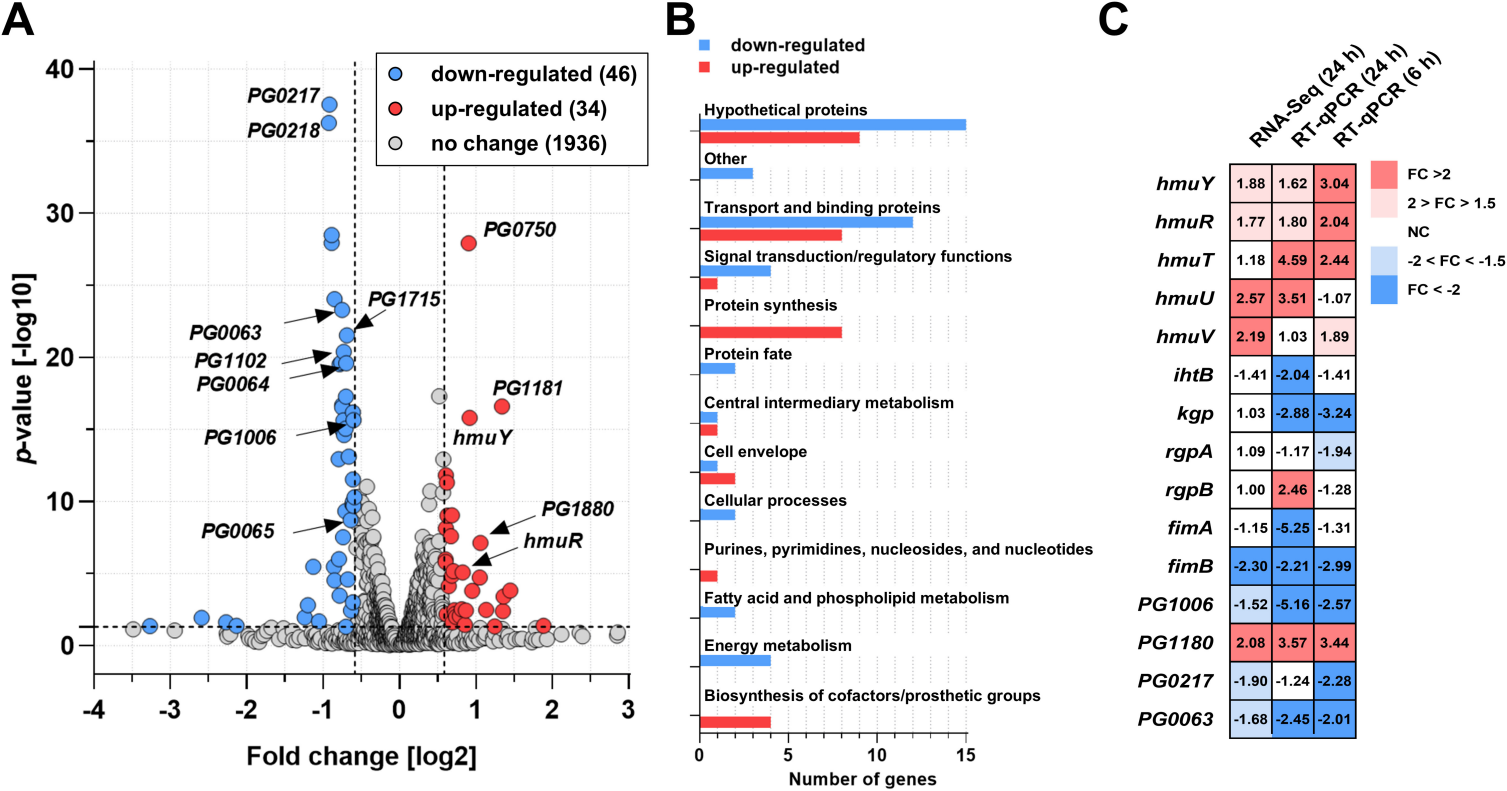
Influence of the *hmuS^Pg^* gene deletion on the expression of other genes. **(A)** Global gene expression was analyzed using mRNA sequencing (RNA-seq analysis). The volcano plot illustrates the distribution of gene expression changes following the deletion of the *hmuS^Pg^* gene in the wild-type strain. Statistically significant changes were defined when a fold change of < 0.667 (log_2_-0.585) or > 1.5 (log_2_0.585) and *p*-values < 0.05 (-log_10_1.301) were obtained. Indicated gene names shown in the volcano plot are associated with membrane transport. **(B)** Functional protein groups encoded by significantly altered genes are categorized into up-regulated (red) and down-regulated (blue) groups. **(C)** Expression of chosen genes was confirmed using RT-qPCR. The change in gene expression is presented as a heatmap. Decreases and increases in gene expression are indicated in blue and red, respectively. Analyses are shown for three independent replicates.

To better understand this phenomenon and to determine whether the inactivation of the *hmuS^Pg^* gene influences the expression of other *P. gingivalis* genes, global mRNA levels were assessed using RNA-seq analysis in bacteria grown for 24 h in Hm medium (Figures 6A–C and Supplementary Table S3). The deletion of the *hmuS^Pg^* gene affected the expression of 80 genes, resulting in decreased and increased transcripts for 46 and 34 genes, respectively (Figure 6A). Among genes whose expression changed in the Δ*hmuS^Pg^* mutant strain, there are two main groups of genes encoding transport and binding proteins and hypothetical or unknown proteins (Figure 6B and Supplementary Table S3). Due to the predicted role of the HmuS^Pg^ and RNA-seq data, in RT-qPCR, we focused on genes involved mainly in iron and heme transport and their homeostasis (Figure 6C and Supplementary Table S3).

**FIGURE 7:**
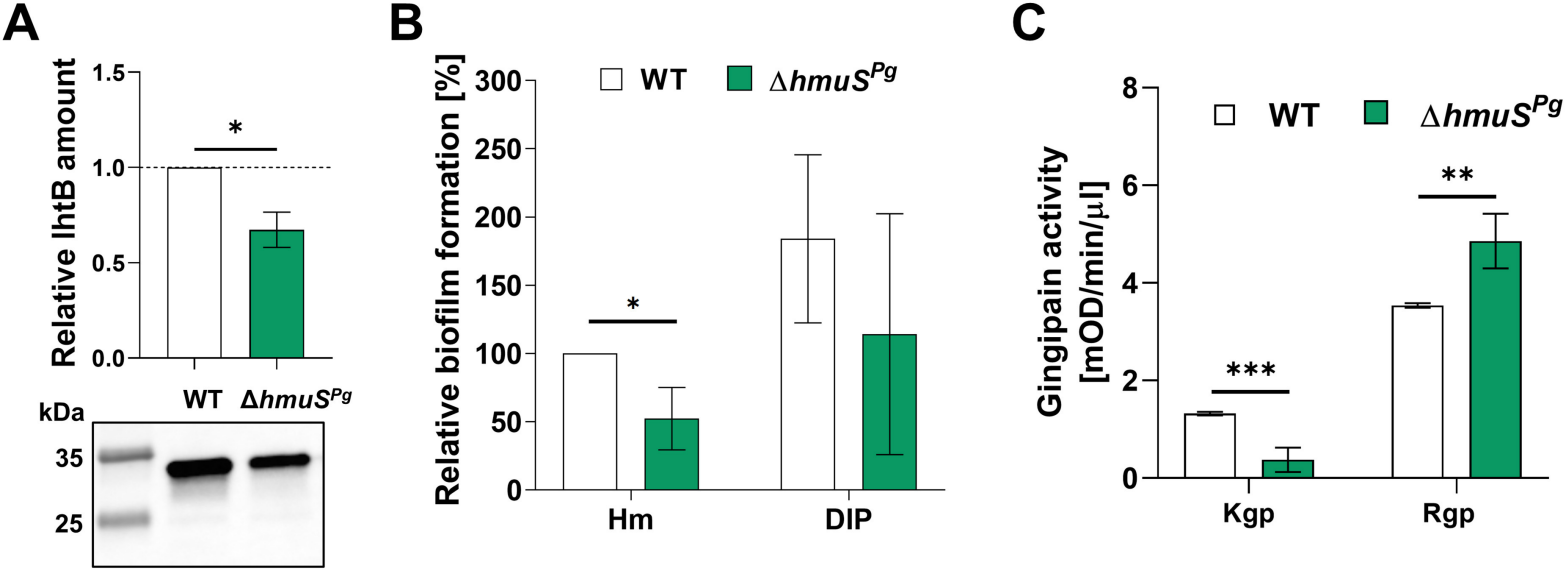
Phenotypic characterization of *Porphyromonas gingivalis* strains. **(A)** The production of IhtB protein was analyzed in whole bacterial cultures grown for 24 h under heme-rich conditions using Western blotting. **(B)** Biofilm formation on an abiotic surface was determined after growing bacteria on polystyrene plates for 48 h under heme-rich conditions (Hm) or after starvation from iron and heme (DIP). Biofilm formation is shown relative to the wild-type (WT) strain grown under Hm conditions (set as 100%). **(C)** The enzymatic activity of Kgp and Rgp gingipains was determined using designated substrates in whole bacterial cultures grown for 24 h under heme-rich conditions. Analyses are shown for three **(A)**, six **(B)**, and four **(C)** replicates, and values are reported as mean ± standard deviation. *p < 0.05; **p < 0.01; ***p < 0.001.

Among genes whose expression increased in the Δ*hmuS^Pg^* mutant strain are those encoding other Hmu system proteins and PG1178-PG1180 proteins (a part of the putative transport system; PG1181-PG1177), and outer membrane transport proteins (Figure 6C and Supplementary Table S3). On the contrary, the Δ*hmuS^Pg^* mutant strain is characterized by a decrease in the expression of genes encoding other transport proteins, including TonB-dependent receptors (PG1006 and PG1715), membrane beta-barrel domain-containing proteins (PG0217 and PG0218, encoded on *PG0214-PG0218* operon), PG1304 (beta-barrel domain-containing protein), and a putative cation efflux system encoded on *PG0063-PG0065* operon (Figure 6C and Supplementary Table S3). Interestingly, expression of the *ihtB* gene, encoding putative ferrochelatase/reverse ferrochelatase, was lower in the Δ*hmuS^Pg^* mutant strain, which was mostly seen in RT-qPCR analysis (Figure 6C) and confirmed at the protein level (Figure 7A).

As shown in the transcriptomic results, ion/heme homeostasis is likely altered in the Δ*hmuS^Pg^* mutant strain, a change that may drive remodeling of bacterial virulence potential. Moreover, the Δ*hmuS^Pg^* mutant strain is characterized by lower expression of genes encoding FimA and FimB proteins (a building block of the long fimbriae) (Figure 6C and Supplementary Table S3). Therefore, biofilm formation was analyzed. As compared to the wild-type strain, the Δ*hmuS^Pg^* mutant strain formed smaller biofilm structures on an abiotic surface under heme-replete conditions (Hm medium) (Figure 7B). Under heme and iron starvation (DIP medium), although a tendency toward reduced biofilm formation was observed in the case of the Δ*hmuS^Pg^* mutant strain, variable results were obtained, with no statistically significant differences (Figure 7B).

Although no changes were detected with RNA-seq analysis in the expression of genes encoding gingipains (*rgpA*, *rgpB*, and *kgp*), these proteolytic enzymes are among the most important virulence factors produced by *P. gingivalis*. Gingipains are involved in nutrient acquisition, including heme. Furthermore, RgpA and Kgp contain hemagglutinin domains that mediate interactions between *P. gingivalis* and other bacteria or host cells (Śmiga and Olczak, 2025a). Therefore, the influence of *hmuS^Pg^* gene deletion on gingipain expression at mRNA and protein levels was analyzed in more detail. RT-qPCR analysis of transcripts showed a lower level of *kgp* mRNA (Figure 6C and Supplementary Table S3), which correlated with lower Kgp activity in whole bacterial cultures of the Δ*hmuS^Pg^* mutant strain, as compared to the wild-type strain (Figure 7C). Slightly lower *rgpA* mRNA levels after 6 h and higher *rgpB* mRNA levels after 24 h were determined in the Δ*hmuS^Pg^* mutant strain compared to the wild-type strain (Figure 6C). However, Rgp enzymatic activity, which represents the activity of both RgpA and RgpB, was higher in the Δ*hmuS^Pg^* mutant strain (Figure 7C). Altogether, inactivation of the *hmuS^Pg^* gene may suggest altered virulence-associated protease activity.

## 4 Discussion

Bacteria often use siderophores to obtain iron. However, host-associated members of the Bacteroidota phylum do not produce siderophores, and only some of them can acquire iron chelated by xenosiderophores (siderophores produced by other microorganisms). Among them are *B. fragilis*, utilizing Fe(III)-ferrichrome, or *Bacteroides vulgatus* and *Bacteroides thetaiotaomicron*, utilizing both Fe(III)-enterobactin and Fe(III)-salmochelin S4 (Rocha and Krykunivsky, 2017). *P. gingivalis* neither synthesizes siderophores nor utilizes xenosiderophores; therefore, as a heme auxotroph, it relies mainly on heme as an iron source. It has been suggested that, when *B. fragilis* grows in an environment where heme is the main source of iron, the iron may be released from heme due to the reverse ferrochelatase activity of the BtuS1 and HmuS^Bf^ (BtuS2) proteins and further transported through the inner membrane by the FeoAB system (Rocha et al., 2019). It has been further proposed that, although *B. thetaiotaomicron* encodes only one HmuS protein homolog (HmuS^Bt^), the bacterium can use a similar mechanism to obtain iron from heme (Meslé et al., 2023; Nath et al., 2025). Therefore, we assumed that HmuS^Pg^ may extract iron from heme, providing *P. gingivalis* with this element. It is worth noting here that its natural environment is often characterized, in contrast to the gut, by lower heme availability. *P. gingivalis* encodes two FeoB proteins, homologous to *E. coli* and *B. fragilis* proteins, potentially responsible for iron or manganese transport through the inner membrane (Nelson et al., 2003; Dashper et al., 2005; He et al., 2006; Anaya-Bergman et al., 2010; Lewis, 2010; Zhang et al., 2016; Rocha et al., 2019). Expression of both *P. gingivalis feoB* genes increases under iron- and heme-limited conditions (Śmiga et al., 2024a,b), suggesting their role in acquiring iron to support bacterial survival and growth in heme-restricted environments. Therefore, similar to *B. fragilis*, *P. gingivalis* FeoB might transport iron released in the periplasmic space from heme by the HmuS^Pg^ protein.

The HmuS^Pg^ protein is encoded within the *hmu* operon, a component of the *P. gingivalis* core genome (Brunner et al., 2010). In addition, the *hmuS^Pg^* gene was found to be preferentially conserved primarily among highly invasive strains, suggesting that it may represent an important virulence-associated factor (Brunner et al., 2010). Importantly, the genes of the *hmu* operon in many Bacteroidota members, including *P. gingivalis*, *B. fragilis*, and *B. thetaiotaomicron*, are upregulated under iron and heme starvation, although at different transcript levels (Lewis et al., 2006; Olczak et al., 2008; Dashper et al., 2009; Lewis and Gui, 2023; Rocha et al., 2019; Nath et al., 2025; and this study). Using Northern blotting and the probe specific for the *hmuS^Pg^* gene, no hybridization signal was visible with RNA isolated from *P. gingivalis* grown in iron/heme-replete or iron/heme-depleted conditions (Lewis et al., 2006), which was in contrast to *hmuY* or *hmuR* probes (Lewis et al., 2006; Olczak et al., 2008). We showed that *hmuS^Pg^* mRNA is produced as a component of the entire *hmuY-hmuV* transcript at extremely low levels, even in bacteria starved of iron and heme (Olczak et al., 2008). This may suggest that the HmuS^Pg^ protein is produced at a very low level under laboratory conditions. Indeed, although we could monitor the *hmuS^Pg^* transcript level, we were not able to detect the production of the HmuS^Pg^ protein, even in the modified strains.

We assigned the HmuS^Pg^ protein to the I class of the chelatase family because it contains a domain characteristic of cobaltochelatases, such as the CobN cobaltochelatase or ChlH magnesium chelatase. Chelatases catalyze the insertion of divalent metal ions into a tetrapyrrole ring (Al-Karadaghi et al., 2006; Bryant et al., 2020; Ogawa et al., 2024). They are categorized into three classes based on structural features, including class-specific folds, ATP dependence, and mono- or multifunctionality (Brindley et al., 2003). Importantly, some chelatases may also function as reverse chelatases (Chau et al., 2010; Taketani et al., 2007). *P. gingivalis* HmuS^Pg^ and HmuS proteins of the Bacteroides species, similar to the CobN and ChlH, are large proteins composed of 6 domains, allowing functioning in the form of monomers. However, it is worth noting that the HmuS^Pg^ exhibits distinct features as compared to classical chelatases.

HmuS^Pg^ binds heme with an ability comparable to that exhibited by *B. fragilis* HmuS^Bf^. Similar to HmuS proteins from *Bacteroides* species (Rocha et al., 2019; Nath et al., 2025), HmuS^Pg^ may be engaged in heme metabolism. The inactivation of the *hmuS^Pg^* gene resulted in a smaller amount of PPIX, determined in the bacterial cells and pigment formed on the surface of the Δ*hmuS^Pg^* mutant strain, suggesting it may have lost the ability to release iron from heme and produce PPIX. Analysis of HmuS protein structures revealed in the *P. gingivalis* HmuS^Pg^ protein the putative accommodation of two heme-binding sites, with Met75 and His252 (site I) and His544 and His1217 (site II). Both sites are conserved within the HmuS proteins among the Bacteroidota phylum, indicating their potential role in iron removal from heme through a potential stepwise mechanism of heme binding (site I) and iron extraction from the PPIX ring (site II). Recently, it has been shown that His538 of HmuS^Bt^ (His544 in HmuS^Pg^) does not bind heme but is involved in its enzymatic activity and may form a metal-binding site with His1209 (His1217 in HmuS^Pg^) (Nath et al., 2025).

*In vitro* activity of the HmuS^Pg^ was stimulated by the inner membrane fraction obtained from *P. gingivalis*, and with much lower efficiency, by the inner membrane fraction obtained from *E. coli* cells. Lipids likely influence the activity of the HmuS^Pg^, similarly to HmuS homolog from *B. thetaiotaomicron* (Nath et al., 2025), and forward and reverse ferrochelatases (Mazanowska et al., 1966; Simpson and Poulson, 1977; Cánepa and Llambías, 1988; Chau et al., 2010). We hypothesize that the inner membrane fraction may be required to stabilize the active conformation of the HmuS^Pg^ protein or form a functional heme-processing complex. Lipid components present in the native inner membrane fraction may be necessary to maintain the protein conformation/protein positioning towards heme (and therefore affect its function), because *in vivo* it is associated with lipids. We also cannot exclude the possibility that other components of the Hmu system, namely HmuT (putative permease), HmuU (MotA/TolQ/ExbB family protein), and HmuV (hypothetical protein), may affect this activity. This suggests that, although differences exist between species, HmuS proteins within the Bacteroidota phylum may play a similar function.

The NADH dependence of HmuS^Pg^ activity demonstrated in our study refers to *in vitro* conditions and does not imply that NADH directly serves as the physiological electron donor in the periplasmic space. A similar uncertainty was noted for the HmuS^Bt^ homolog from *B. thetaiotaomicron* (Nath et al., 2025), in which HmuS^Bt^ activity was also observed under reducing conditions. However, the authors emphasized that the *in vivo* electron-transfer mechanism remains unresolved and may involve redox intermediates, such as flavodoxins or membrane-associated electron-transport components. Likewise, in *B. fragilis*, Rocha et al. (2019) supported a model of periplasmic iron release from heme employing membrane-associated proteins, but without identifying a specific physiological electron donor. In this context, it appears most plausible that, under cellular conditions, HmuS^Pg^ may be functionally coupled to membrane-associated redox components that mediate electron transfer to the periplasmically exposed active site.

We are aware that in the absence of a direct quantitative assessment of iron and demonstrated catalytic turnover, our data do not unequivocally prove catalytic removal of iron. Although the detection of heme and PPIX in the wild-type and mutant strains shows the involvement of HmuS^Pg^ in heme metabolism, we were unable to measure the levels of reaction products in the *in vitro* reaction. However, a decrease in the PPIX levels in bacterial cells and pigment formed on the surface of the Δ*hmuS^Pg^* mutant strain may suggest the engagement of the HmuS^Pg^ in iron removal from heme.

As compared to HmuS proteins of *B. fragilis* and *B. thetaiotaomicron* (Rocha et al., 2019; Nath et al., 2025), deletion of the *hmuS^Pg^* gene did not significantly influence the *P. gingivalis* phenotype examined under laboratory conditions. It is likely that when bacteria grow in culture media in the form of monoculture, the product of the *hmuS^Pg^* gene is not crucial, because other alternative systems (e.g., Iht system or other, so far not identified mechanism) may fulfill the need for iron in *P. gingivalis* (Smalley and Olczak, 2017; Śmiga and Olczak, 2025a). Moreover, in contrast to Bacteroides species, *P. gingivalis* produces a functional HemH protein (Śmiga et al., 2026), which might be important to maintain balance between iron and heme inside the bacterial cell, therefore contributing to the lower impact of the *hmuS^Pg^* gene inactivation on *P. gingivalis* phenotype.

Inactivation of the *hmuS^Pg^* gene resulted in changes in the expression of genes whose products, among others, are involved in transport and binding. This includes increased expression of genes encoded on the *hmu* operon, suggesting a compensatory effect due to increased requirement of these proteins for heme delivery to fulfill the iron needs. On the other hand, the expression of genes encoded on the *PG0063-PG0065* operon, assigned as putative cation efflux systems, could suggest a disruption of ion homeostasis, most likely involving iron.

Biofilm formation is tightly regulated in *P. gingivalis* by heme availability, with heme limitation promoting the development of greater biofilm structures (Śmiga et al., 2024a). Therefore, disrupted iron or heme homeostasis of the Δ*hmuS^Pg^* mutant strain may partly explain the reduced expression of *fimA* and *fimB* genes, which encode a component of long fimbriae, although Fim proteins are less expressed in the W83 strain. Consequently, reduced biofilm-forming capacity in monocultures on abiotic surfaces might be associated with deletion of the *hmuS^Pg^* gene. Furthermore, disruption of iron and heme homeostasis may contribute to changes in gingipain activity and the reduced production of the IhtB protein, the latter likely involved in iron acquisition from heme (Śmiga and Olczak, 2025a). Increased Rgp activity may enhance the proteolytic destabilization of hemoglobin and promote the conversion to methemoglobin, thereby potentially increasing the rate of heme release. In contrast, reduced Kgp activity could impair subsequent processing and stabilization of released heme at the bacterial surface, leading to an altered balance between heme release and its effective capture or utilization rather than a straightforward increase in heme availability.

**FIGURE 8:**
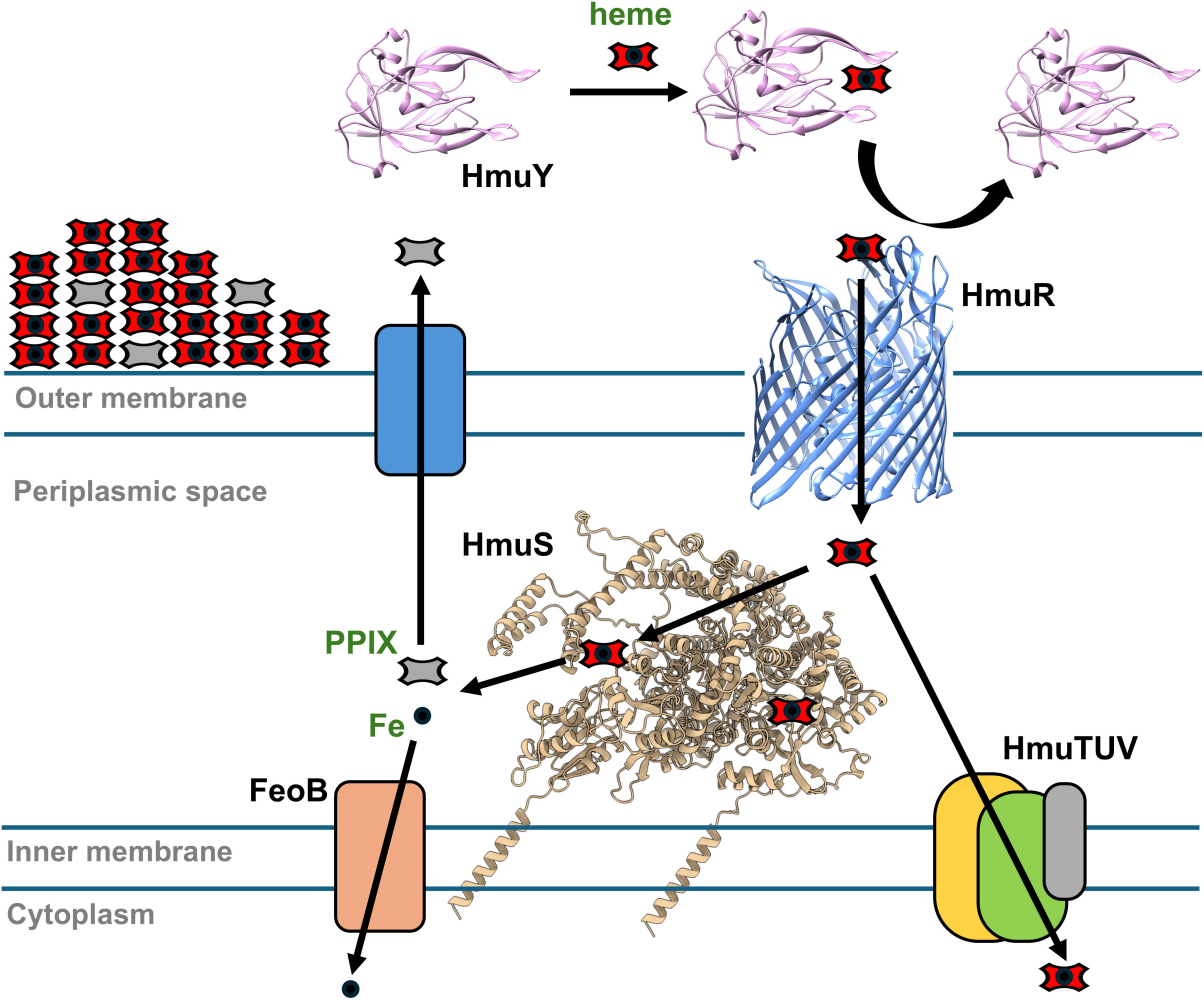
Schematic presentation of iron acquisition with the engagement of the HmuS^Pg^. *Porphyromonas gingivalis* uses the Hmu system to obtain heme, which can also be an iron source. HmuY hemophore-like protein captures heme from host hemoproteins and delivers it to HmuR TonB-dependent outer membrane receptor for transport to the periplasmic space. HmuTUV proteins are presumably involved in transporting heme across the inner membrane. HmuS participates in heme metabolism to release iron in the periplasmic space and produce PPIX. Iron is transported into the cytoplasm via FeoB. PPIX can be exported and deposited on the cell surface, forming a component of heme-based pigment.

In conclusion, *P. gingivalis* HmuS^Pg^ *in vitro* binds heme and exhibits activity, which suggests its engagement in heme metabolism. The reaction requires a hydride donor and components of the inner membrane fraction of *P. gingivalis* cells. *In vivo* analyses show iron removal from the heme and PPIX production. The resulting iron-free PPIX may either be exported and deposited on the bacterial surface as a pigment component or transported into the cytoplasm, while iron is likely imported into the cytoplasm *via* the FeoB transport system (Figure 8). At present, it remains unclear whether the PPIX ring undergoes further degradation, as has been reported for the HmuS protein from *Yersinia pseudotuberculosis* (Onzuka et al., 2017). Considering inconsistencies in results obtained from experiments performed *in vitro* and *in vivo*, it cannot be ruled out that the HmuS^Pg^ may have additional functions depending on the availability of heme and iron. HmuS^Pg^ binds heme and may contribute to the overall activity of the Hmu system, participating in heme metabolism as a component of the heme-processing complex (Figure 8). Similar to proteins described in *E. coli* K-12 (Létoffé et al., 2009), HmuS^Pg^ could act as a heme chaperone to avoid heme toxicity. However, most probably, in the absence of a typical periplasmic binding protein in the *P. gingivalis* Hmu system, HmuS^Pg^ might transfer heme from HmuR to the inner membrane complex (HmuTUV) (Figure 8). Importantly, these potential mechanisms may depend on environmental conditions and could be utilized by *P. gingivalis in vivo* under specific, yet undefined, states of iron demand, particularly when the bacterium grows within deeper layers of the biofilm or inside host cells. Data presented here on HmuS^Pg^ and its homolog from *B. fragilis*, as well as those reported on the homolog from *B. thetaiotaomicron* (Nath et al., 2025), indicate that the HmuS protein family represents a novel mechanism for heme utilization. Nevertheless, further studies will be necessary to fully elucidate the physiological role and mechanistic functions of HmuS in *P. gingivalis* and other members of the Bacteoidota phylum.

## Data Availability

The dataset is publicly available at https://www.ebi.ac.uk/biostudies/ArrayExpress/studies/E-MTAB-16197, and can be accessed using the accession code E-MTAB-16197 in the ArrayExpress repository.

## Footnotes

1
https://skyline.ms/project/home/software/skyline/begin.view

2
https://www.ncbi.nlm.nih.gov/protein

3
https://alphafold.com

4
https://www.cgl.ucsf.edu/chimera
