# Supplementary material for: Biochemical and functional characterization of Porphyromonas gingivalis HmuS protein reveals its participation in heme metabolism
Source: Front Microbiol. 2026 May 19;17:1788076. doi: 10.3389/fmicb.2026.1788076 (PMC13227078; doi:10.3389/fmicb.2026.1788076)
Supplement: Supplementary file 2 [file Data_Sheet_1.pdf]

Supplementary material for:

Biochemical and functional characterization of *Porphyromonas gingivalis* HmuS protein reveals its participation in heme metabolism

Patryk Cierpisz[1], Michał Śmiga[1,*], Michał Tracz[2], Ronivaldo Rodrigues da Silva[3], Jennifer L. DuBois[3], Teresa Olczak[1*]

[1]Laboratory of Medical Biology, Faculty of Biotechnology, University of Wrocław, 50-383 Wrocław, Poland
[2]Laboratory of Mass Spectrometry, Faculty of Biotechnology, University of Wrocław, 50-383 Wrocław, Poland
[3]Department of Chemistry and Biochemistry, Montana State University, Bozeman 59717, MT, USA

*Corresponding authors: michal.smiga@uwr.edu.pl, teresa.olczak@uwr.edu.pl

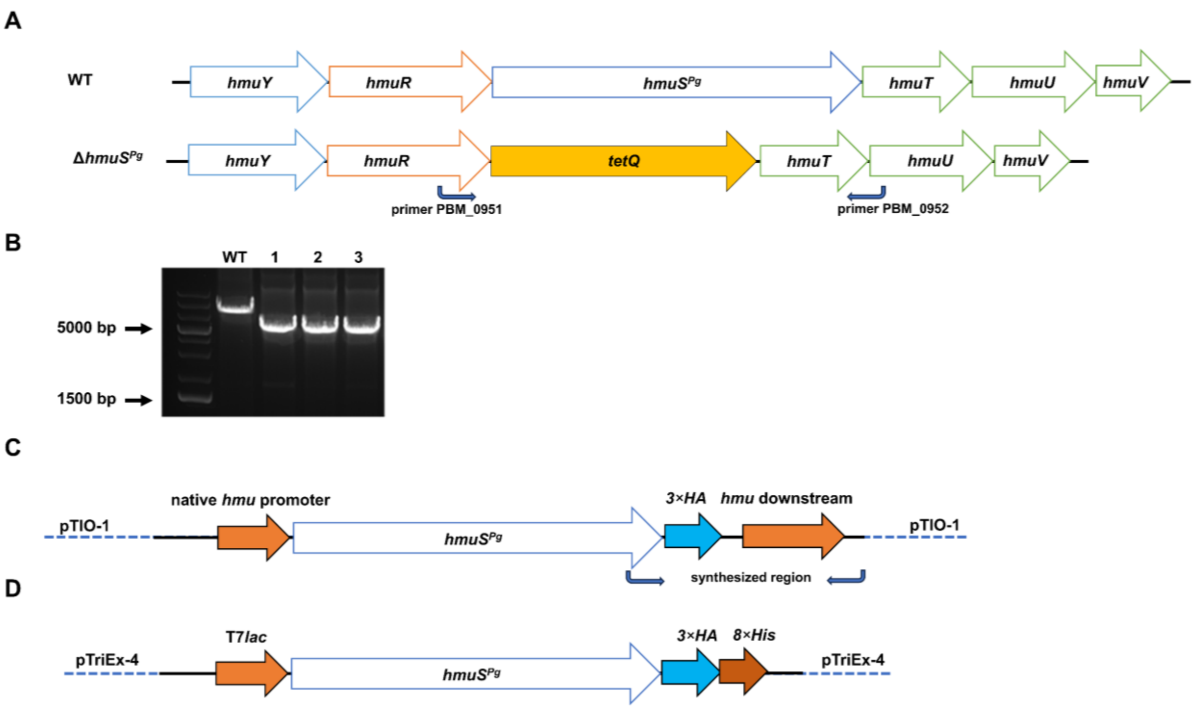

Figure S1. Schematic presentation of modified *Porphyromonas gingivalis* strains and constructed plasmids. (A) Construction of the Δ*hmuS*^{Pg} mutant strain. (B) Verification of the mutation using PCR. Genomic DNA of the wild-type (WT) and Δ*hmuS*^{Pg} mutant (clones 1-3) strains was used as a template for PCR. Schematic presentation of PCR-amplified fragments using genomic DNA with primer names shown in (A). (C) Construction of WT+HmuS^{Pg}-HA control and Δ*hmuS*^{Pg}+HmuS^{Pg}-HA complemented strains using the pTIO-1 plasmid, containing the *hmuS*^{Pg} gene with the *hmu* promoter. (D) Construction of the plasmid expressing HmuS^{Pg}-HA-His protein.

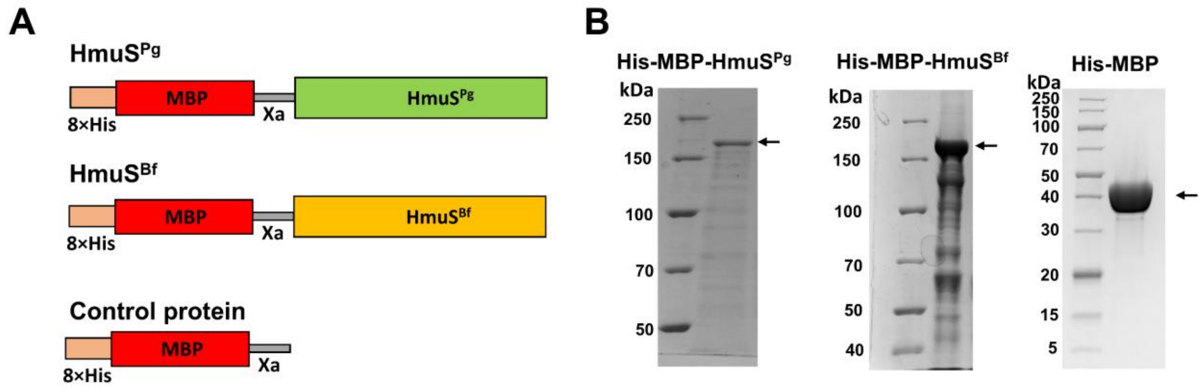

Figure S2. Schematic presentation of proteins purified and analyzed in this study (A). Representative purified recombinant *Porphyromonas gingivalis* HmuS (His-MBP-HmuS^{Pg}), *Bacteroides fragilis* HmuS (His-MBP-HmuS^{Bf}), and control His-MBP protein samples (indicated by arrows) were separated by SDS-PAGE and stained with CBB G-250 (B).

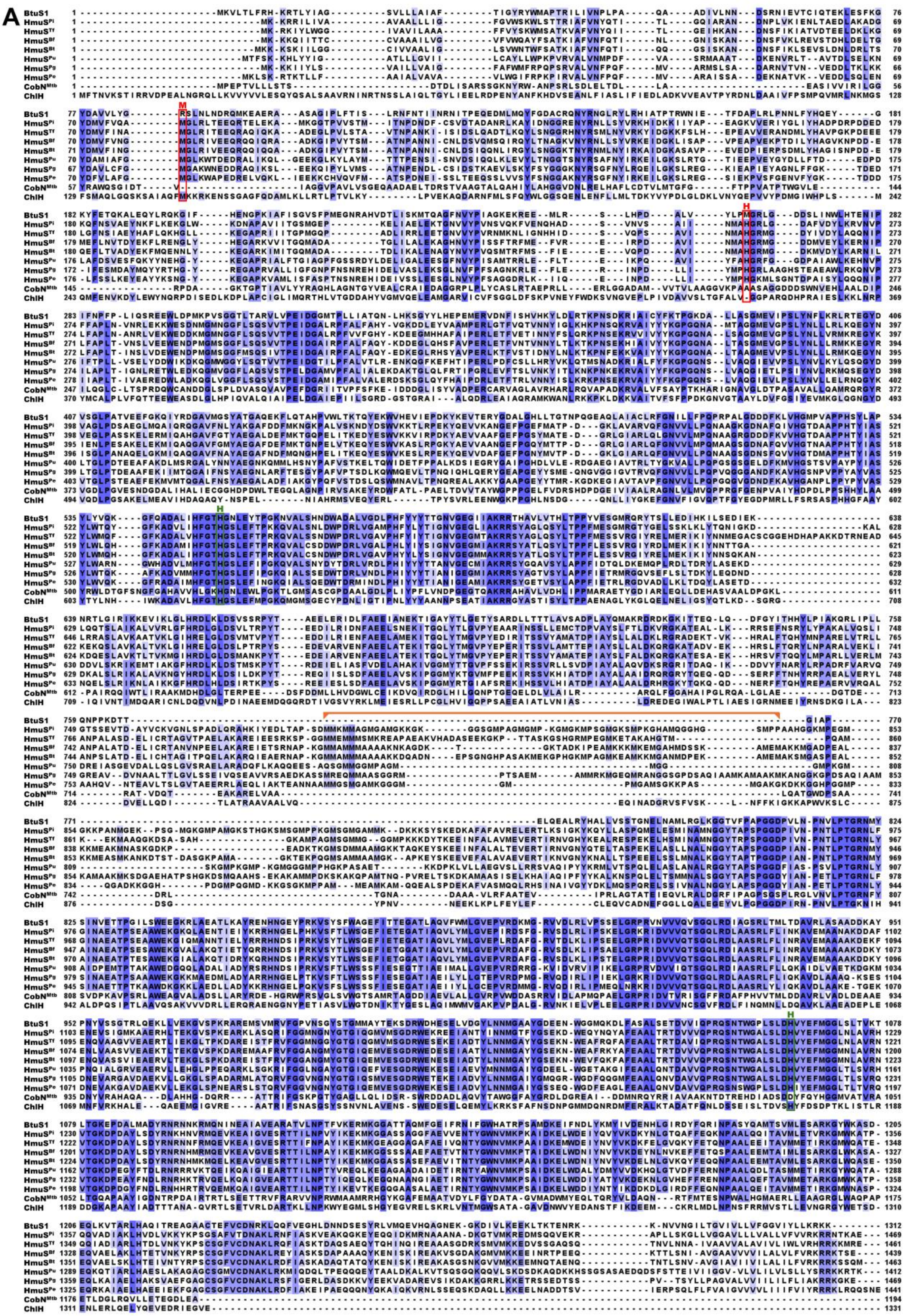

B

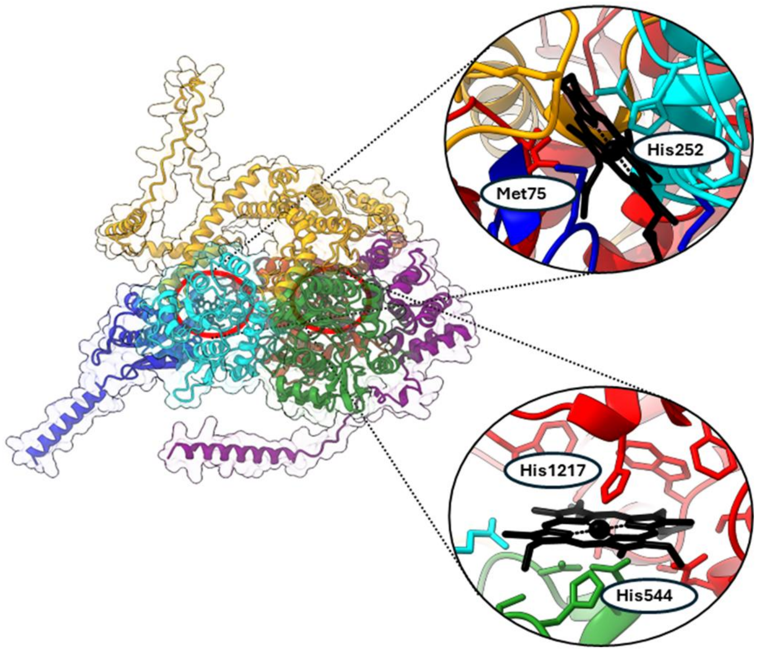

Figure S3. (A) Alignment of amino acid sequence of *Porphyromonas gingivalis* $HmuS^{Pg}$ and its homologs from *Bacteroides fragilis* ($HmuS^{Bf}$, BtuS1), *Bacteroides thetaiotaomicron* ($HmuS^{Bt}$), *Prevotella intermedia* ($HmuS^{Pi}$), *Tannerella forsythia* ($HmuS^{Tf}$), *Porphyromonas endodontalis* ($HmuS^{Pe}$), *Porphyromonas uenonis* ($HmuS^{Pu}$), cobaltochelatase from *Mycobacterium tuberculosis* ($CobN^{Mtb}$), and magnesium chelatase from *Synechocystis sp*. PCC 6803 (ChlH). Identical amino acids are marked in blue. Amino acids potentially involved in heme-iron coordination or ferrochelatase activity, homologous to $HmuS^{Pg}$ Met75 and His252 (I binding site) or His544 and His1217 (II binding site), are indicated by red or green, respectively. The region rich in methionines within the IV domain is marked with an orange bracket. (B) Position of the site I and site II in the $HmuS^{Pg}$ protein structure model. The theoretical analyses were performed using AlphaFold 3.0 structural modeling. Using the $HmuS^{Pg}$ amino acid sequence, the protein structure was modeled in the presence of two heme ligands.

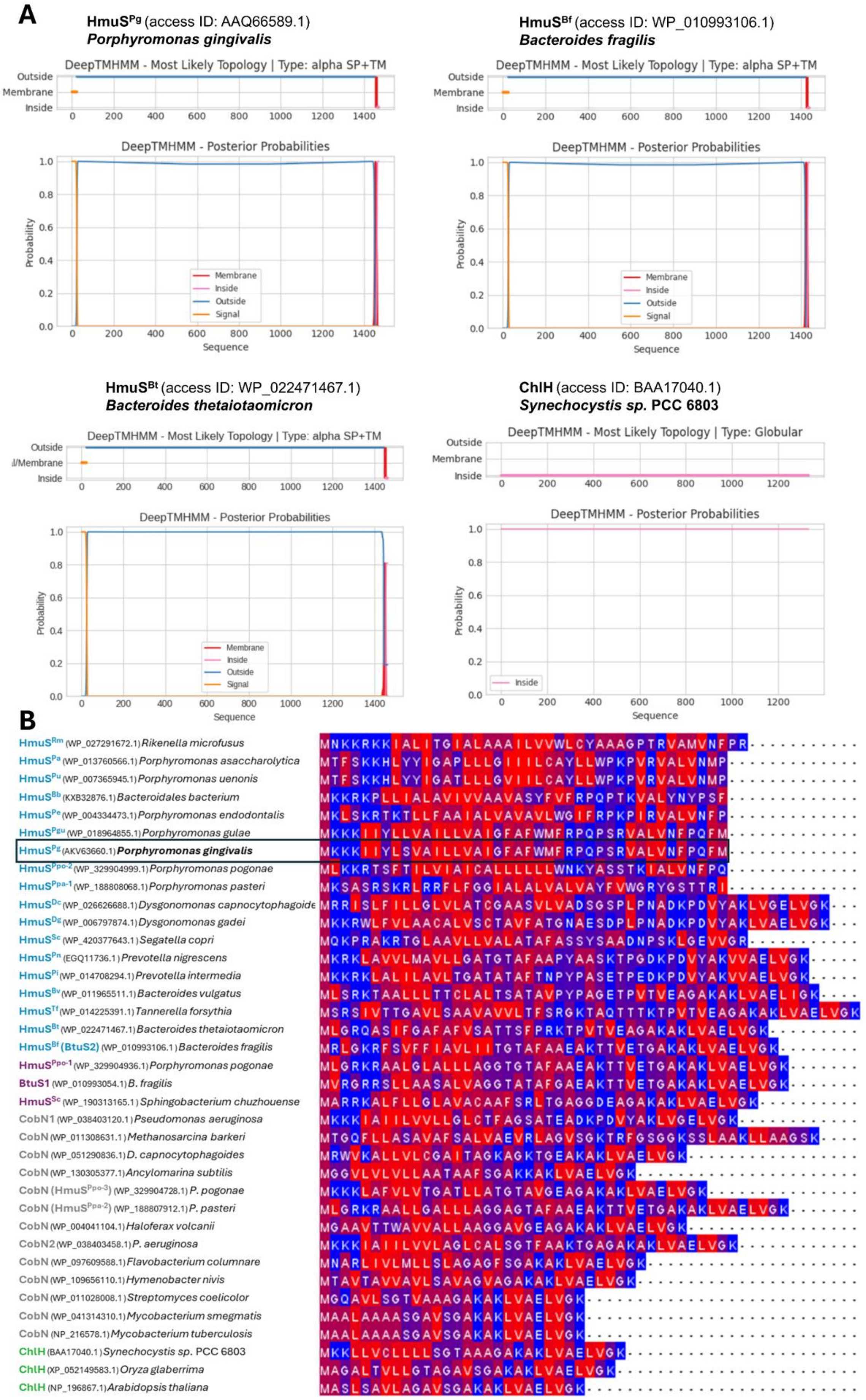
A
HmuS^{Pg} (access ID: AAQ66589.1)
Porphyromonas gingivalis
HmuS^{Bf} (access ID: WP_010993106.1)
Bacteroides fragilis
HmuS^{Bt} (access ID: WP_022471467.1)
Bacteroides thetaiotaomicron
ChlH (access ID: BAA17040.1)
Synechocystis sp. PCC 6803
DeepTMHMM - Most Likely Topology | Type: alpha SP+TM
DeepTMHMM - Most Likely Topology | Type: Globular
DeepTMHMM - Posterior Probabilities
Outside
Membrane
Inside
Signal
Probability
Sequence
B
HmuS^{Rm} (WP_027291672.1) Rikenella microfusus
HmuS^{Pa} (WP_013760566.1) Porphyromonas asaccharolytica
HmuS^{Pu} (WP_007365945.1) Porphyromonas uenonis
HmuS^{Bb} (KXB32876.1) Bacteroidales bacterium
HmuS^{Pe} (WP_004334473.1) Porphyromonas endodontalis
HmuS^{Pgu} (WP_018964855.1) Porphyromonas gulae
HmuS^{Pg} (AKV63660.1) Porphyromonas gingivalis
HmuS^{Ppo-2} (WP_329904999.1) Porphyromonas pogonae
HmuS^{Ppa-1} (WP_188808068.1) Porphyromonas pasteri
HmuS^{Dc} (WP_026626688.1) Dysgonomonas capnocytophagoide
HmuS^{Dg} (WP_006797874.1) Dysgonomonas gadei
HmuS^{Sc} (WP_420377643.1) Segatella copri
HmuS^{Pn} (EGQ11736.1) Prevotella nigrescens
HmuS^{Pi} (WP_014708294.1) Prevotella intermedia
HmuS^{Bv} (WP_011965511.1) Bacteroides vulgatus
HmuS^{Tf} (WP_014225391.1) Tannerella forsythia
HmuS^{Bt} (WP_022471467.1) Bacteroides thetaiotaomicron
HmuS^{Bf} (BtuS2) (WP_010993106.1) Bacteroides fragilis
HmuS^{Ppo-1} (WP_329904936.1) Porphyromonas pogonae
BtuS1 (WP_010993054.1) B. fragilis
HmuS^{Sc} (WP_190313165.1) Sphingobacterium chuzhouense
CobN1 (WP_038403120.1) Pseudomonas aeruginosa
CobN (WP_011308631.1) Methanosarcina barkeri
CobN (WP_051290836.1) D. capnocytophagoides
CobN (WP_130305377.1) Ancylomarina subtilis
CobN (HmuS^{Ppo-3}) (WP_329904728.1) P. pogonae
CobN (HmuS^{Ppa-2}) (WP_188807912.1) P. pasteri
CobN (WP_004041104.1) Haloferax volcanii
CobN2 (WP_038403458.1) P. aeruginosa
CobN (WP_097609588.1) Flavobacterium columnare
CobN (WP_109656110.1) Hymenobacter nivis
CobN (WP_011028008.1) Streptomyces coelicolor
CobN (WP_041314310.1) Mycobacterium smegmatis
CobN (NP_216578.1) Mycobacterium tuberculosis
ChlH (BAA17040.1) Synechocystis sp. PCC 6803
ChlH (XP_052149583.1) Oryza glaberrima
ChlH (NP_196867.1) Arabidopsis thaliana

Figure S4. Theoretical prediction of the topology of HmuS proteins from *Porphyromonas gingivalis* ($HmuS^{Pg}$), *Bacteroides fragilis* ($HmuS^{Bf}$), *Bacteroides thetaiotaomicron* ($HmuS^{Bt}$), and ChlH *Synechocystis sp.* PCC 6803 (A). The figure presents the probability of protein topology. (B) Analysis of signal peptide sequences in selected proteins. In the $HmuS^{Pg}$, MKKKIIYLSVAILLVAIGFAFWMF peptide (a positively charged region followed by approximately 15 strongly hydrophobic residues, mainly I, V, L, A, F, W, Y) is typical for signals targeting proteins to the membrane. The region characteristic of signal peptide cleavage is absent. This suggests that the sequence exhibits features of a signal sequence that is not cleaved off, which is typical of proteins associated with the inner membrane.

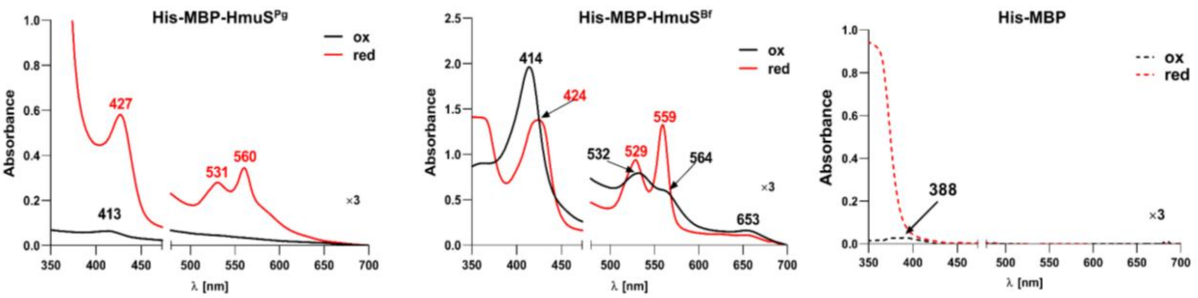

Figure S5. UV-visible spectra of concentrated 55 µM protein samples purified from *Escherichia coli* cells, recorded under oxidizing (ox) and reducing (red) conditions. Reduction was carried out using 10 mM sodium dithionite, with the samples kept under mineral oil overlay. The experiment was conducted three times, and one representative spectrum is shown.

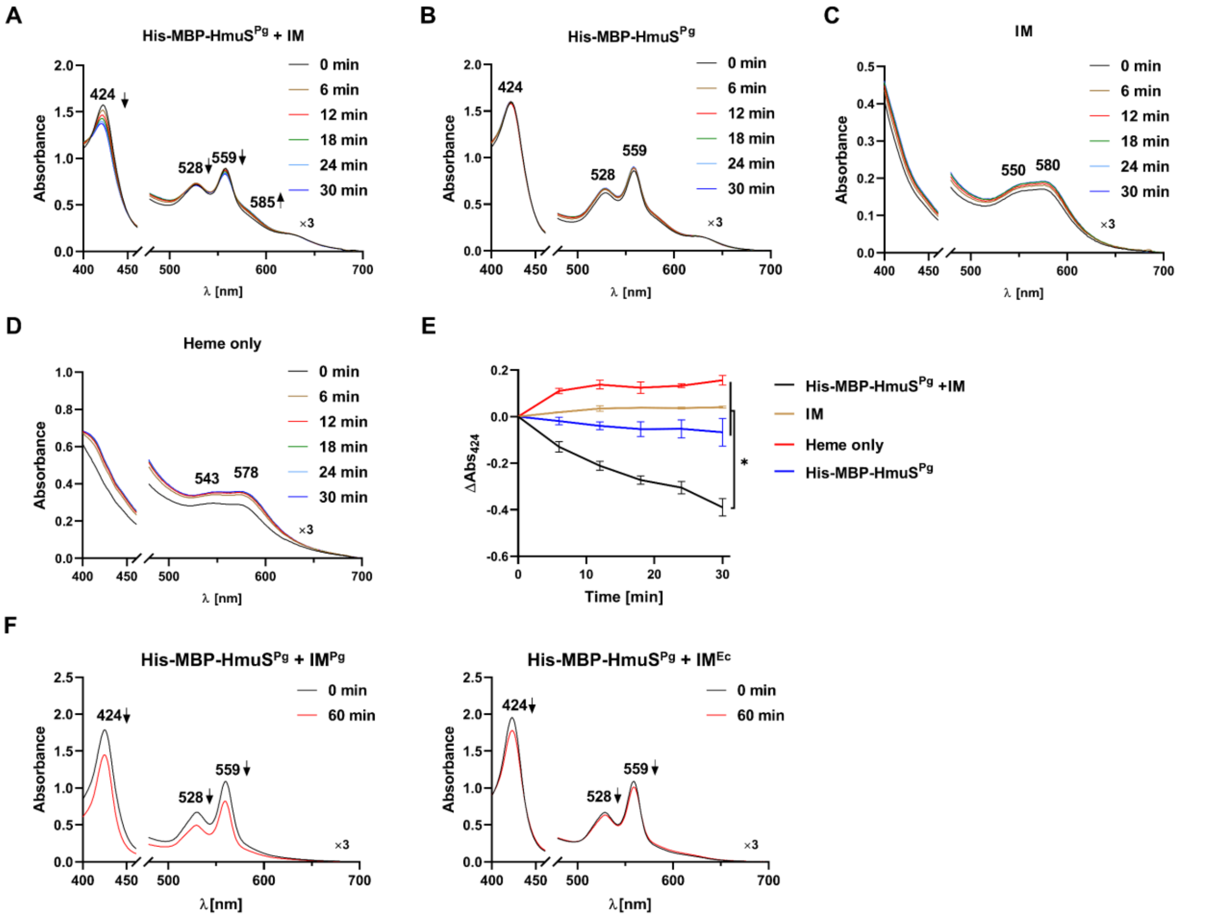

Figure S6. Analysis of heme metabolism. Visible spectra of His-MBP-HmuS^{Pg} with heme, NADH, and inner membrane fraction (IM), the latter isolated from the *Porphyromonas gingivalis* wild-type strain, were analyzed over time (A). The changes in the spectra are indicated with arrows. The control samples contain all the ingredients except IM (B), His-MBP-HmuS^{Pg} (C), or contain heme only (D). The experiment was conducted three times, and representative spectra are shown. (E) A decrease in heme content was determined as the difference in the absorbance at 424 nm (ΔAbs_{424}) at the initial time subtracted from the absorbance at a given time point. Results are shown for three replicates, and values are reported as mean ± standard deviation. *$p < 0.05$. (F) Comparison of heme metabolism in the presence of *P. gingivalis* (IM^{Pg}) or *Escherichia coli* (IM^{Ec}) inner membrane fraction.

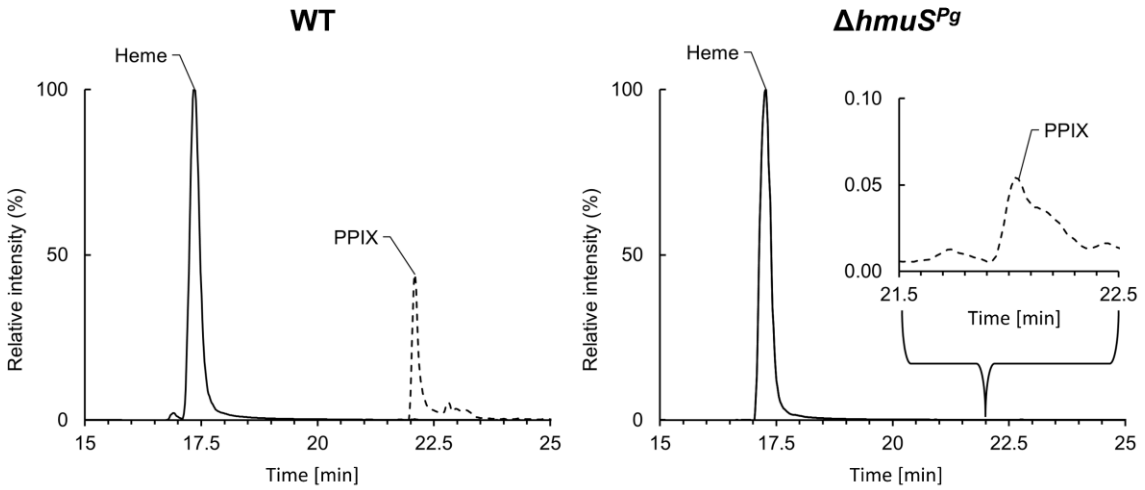

Compound	RT (min)	m/z (most intense isotype/monoisotopic)	Adduct
Heme	17.3	616.18/614.18	M+
PPIX	22.1	563.27/563.27	MH+

Figure S7. Analysis of heme and PPIX content in the *Porphyromonas gingivalis* pigment using targeted liquid chromatography-mass spectrometry (LC-MS). A comparison of the extracted ion chromatogram (XIC) profiles for heme and PPIX across the wild-type (WT) and Δ*hmuS*^{Pg} mutant strains' pigment extracts is shown. The XIC m/z's were 616.18 and 563.27 for heme and PPIX, respectively (±0.05 m/z). As the intensity of the PPIX peak is marginal in the pigment extracted from the Δ*hmuS*^{Pg} mutant strain, an inset zooming onto the chromatogram section of interest is presented. Chromatographic and ionization parameters used for targeted LC-MS analysis of PPIX and heme in pigment extracts are shown below the figure. Parameters were presumed by running commercially available standards.

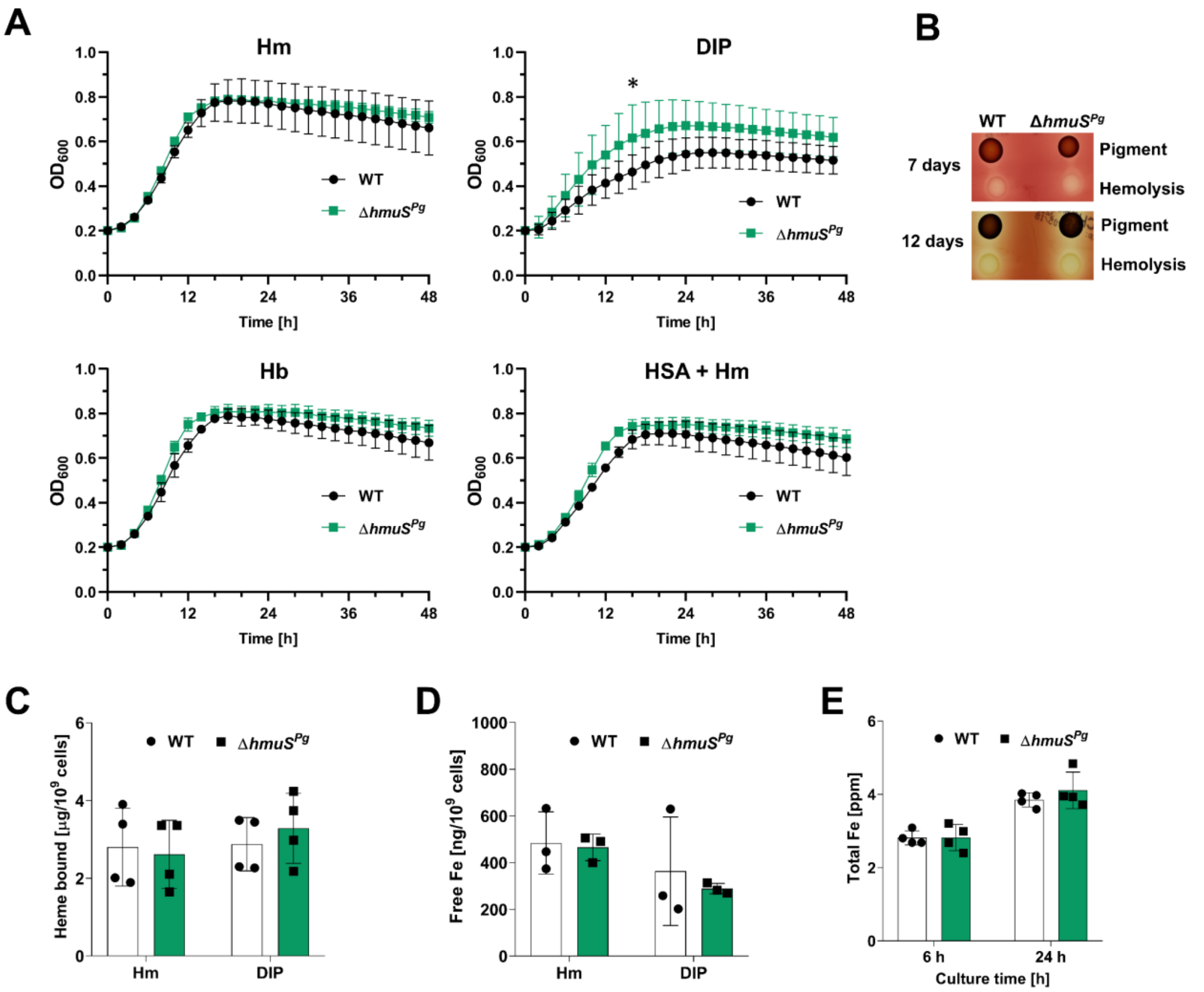

Figure S8. Phenotypic characterization of *Porphyromonas gingivalis* strains. (A) Wild-type (WT) and mutant ($\Delta hmuS^{Pg}$) strains were grown under heme-rich conditions in BM medium supplemented with 7.7 µM heme (Hm), 2 µM hemoglobin (Hb), 7.7 µM human serum albumin with 7.7 µM heme (HSA-Hm), or under iron and heme-limited conditions, using BM without added heme and supplemented with an iron chelator, 160 µM dipyridyl (DIP). Bacterial growth in liquid culture media was monitored over time by measuring optical density at 600 nm (OD_{600}). Results are shown from three independent experiments and are shown as mean±standard error (mean±SE). (B) Bacteria were grown on blood agar plates for 7 and 12 days, and bacterial growth, pigment formation, and hemolytic activity were estimated by visual inspection. Analysis of the $hmuS^{Pg}$ gene deletion on heme and iron homeostasis was investigated by determining heme binding to *P. gingivalis* whole cells (C) and measuring the contents of free iron (D) and total iron (E) in the bacterial cells. Analyses are shown from at least three replicates, and values are shown as mean±standard deviation (mean±SD). *$p<0.05$;

Table S1. List of plasmids generated and used in this study.

Plasmid name	Description	Reference
pTIO-1	plasmid for *P. gingivalis* electroporation, carrying the *ermF* erythromycin resistance cassette	Tagawa et al., 2014
pTIO-tetQ	plasmid for *P. gingivalis* electroporation, carrying the *tetQ* tetracycline resistance cassette	Śmiga et al., 2019
pTIO-$HmuS^{Pg}$	plasmid used to complement the Δ$hmuS^{Pg}$ mutant strain, construct the control strain, and localize $HmuS^{Pg}$ protein in *P. gingivalis*, producing the entire $HmuS^{Pg}$ protein (1-1469 amino acids) with the C-terminal 3×HA tag ($HmuS^{Pg}$-3×HA) under the native *hmu* operon promoter.	this study
pTriEx-$HmuS^{Pg}$	plasmid used for $HmuS^{Pg}$ protein localization in *E. coli*, producing the entire $HmuS^{Pg}$ (1-1469 amino acids) with the C-terminal 3×HA-8×His tag	this study
pMAL-c5xHis	pMAL-c5x plasmid (New England Biolabs) modified by the insertion of the sequence encoding 8×His, resulting in the 8×His tag at the N terminus of the maltose-binding protein (MBP)	Śmiga et al., 2019
pMAL-$HmuS^{Pg}$	plasmid used to overexpress the recombinant $HmuS^{Pg}$ protein, lacking the N-terminal signal peptide sequence (40-1469 amino acids), possessing the N-terminal 8×His-MBP tag	this study
pMAL-$HmuS^{Bf}$	plasmid used to overexpress the $HmuS^{Bf}$ protein, lacking the N-terminal signal peptide sequence (28-1439 amino acids), possessing the N-terminal 8×His-MBP tag	this study
pMAL-$HmuS^{Pg}$_N	plasmid used to overexpress the C-terminally truncated $HmuS^{Pg}$ protein ($HmuS^{Pg}$_N), lacking the N-terminal signal peptide sequence (final protein composed of 40-858 amino acids), possessing the N-terminal 8×His-MBP tag (protein used for rabbit immunization)	this study
pMAL-$HmuS^{Pg}$_C	plasmid used to overexpress the N-terminally truncated $HmuS^{Pg}$ protein ($HmuS^{Pg}$_C; final protein composed of 899-1469 amino acids), possessing the N-terminal 8×His-MBP tag (protein used for rabbit immunization)	this study

Table S2. List of primers and other DNA sequences designed and used in this study.

Primer name	5′→3′ DNA sequence	Gene abbreviation/locus ID	Description (reference)
PBM_024	tcgggatcgagggaaggATGGTGGCACGAATGAGTCTTTC	*hmuS*^{Pg}/PG1553	Amplify DNA sequence encoding HmuS^{Pg} protein, lacking the N-terminal signal peptide sequence (40-1469 amino acid residues), containing the overlapping sequences (indicated with small letters) to the pMAL-c5xHis plasmid digested with XmnI and BamHI restriction enzymes (this study)
PBM_014	tgcagggaattcggatccTCATTCTTTCCCTTTTCTGCGT		
PBM_0897	tacaatcaaaggagatatacATGAAGAAAAAAATAATTTATC	*hmuS*^{Pg}/PG1553	Amplify the entire DNA sequence encoding HmuS^{Pg} protein (1-1469 amino acid residues) to clone the gene into the pTriEx-4 plasmid digested with NcoI and XhoI restriction enzymes (overlapping sequences indicated with small letters) and obtain the protein with C-terminal 3×HA-8×His tag (this study)
PBM_0899	atggtgatggtggtgctcgacGGCATAGTCTGGCACATCATAAG		
PBM_014	tgcagggaattcggatccTCATTCTTTCCCTTTTCTGCGT		
PBM_095	gggatcgagggaaggATGAGTGCCACGAAGATTGC	*hmuS*^{Bf} (*btuS2*)/BF9343_2624	Amplify DNA sequence encoding HmuS^{Bf}, lacking the N-terminal signal peptide sequence (28-1439 amino acid residues), containing the overlapping sequences (indicated with small letters) to the pMAL-c5xHis plasmid digested with XmnI and BamHI restriction enzymes (this study)
PBM_096	cctgcagggaattcgTTACTTTTGCTCTTCCGGTGTCCGGT		
PBM_024	tcgggatcgagggaaggATGGTGGCACGAATGAGTCTTTC	*hmuS*^{Pg}/PG1553	Amplify DNA sequence encoding C-terminally truncated HmuS^{Pg} protein (40-899 amino acid residues), containing overlapping sequences (indicated with small letters) to the pMAL-c5xHis plasmid digested with XmnI and BamHI restriction enzymes (this study)
PBM_021	tgcagggaattcggatccTTAGGCCGGTTGCTTGGCTTTC		
PBM_008	tcgggatcgagggaaggATGACGAATCAGCCAGTACGAG	*hmuS*^{Pg}/PG1553	Amplify DNA sequence encoding N-terminally truncated HmuS^{Pg} protein (899-1469 amino acid residues), containing overlapping sequences (overlapping sequences indicated with small letters) to the pMAL-c5xHis plasmid digested with XmnI and BamHI restriction enzymes (this study)
PBM_014	tgcagggaattcggatccTCATTCTTTCCCTTTTCTGCGT		
PBM_0945	gacaaggcaagggaagaacTCGAAAGAAATGAGAATATTCGGC	*hmuR*/PG1552	Amplify DNA sequence encompassing: 3' region of the *hmuR* gene, *tetQ* antibiotic cassette, and the region encompassing the entire *hmuT* gene and 3' region of the *hmuU* gene, all containing overlapping sequences to respective neighboring genes or the pTIO-1 plasmid (indicated with small letters) to generate the Δ*hmuS*^{Pg} mutant strain in the *P. gingivalis* wild-type W83 strain (this study)
PBM_0946	gaattcctaaatttataatattcatCGTGGCGAATTATATTTCTGTTGC		
PBM_0947	gcaacagaaatataattcgccacgTGAATATTATAAATTTAGGAATTCTTGCTCAC	*tetQ*	
PBM_0948	tcagcaggacttctctcatTTATTTTGATGACATTGATTTTTGGAAC		

PBM_0949	caaaaatcaatgtcatcaaaataaATGAGAGAAGTCCTG CTGA	*hmuT*/PG1554, *hmuU*/PG1555	
PBM_0950	gctctagaactagtggatcAAAGTATTGTTAGTGAGT GGTTCG		
PBM_0951	TCGAAAGAAATGAGAATATTCGGC	*hmuR*/PG1552 *hmuU*/PG1555	Amplify the DNA sequence encompassing the 3' region of the *hmuR* gene and 5' of the *hmuU* gene, used to verify the construction of the Δ*hmuS*^{Pg} mutant strain by PCR and DNA sequencing (this study)
PBM_0952	AAAGTATTGTTAGTGAGTGGTTCG		
PBM_0741	CTTGACTTCAGTGGCGCAG	*16S rRNA/CF003_16SR1*	Used to analyze gene expression by RT-qPCR (Maeda et al., 2003)
PBM_0742	AGGGAAGACGGTTTTCACCA		
PBM_0739	GCTTCGAAATACGAAACGTG	*hmuY*/PG1551	Used to analyze gene expression by RT-qPCR (Gmiterek et al., 2013)
PBM_0740	TATATCCGTCTGTCGGAACG		
PBM_0747	CTACCGACACCATCGTATCC	*hmuR*/PG1552	Used to analyze gene expression by RT-qPCR (Śmiga et al., 2019)
PBM_0748	CATTGAGCTGATCTCTGGAAC		
PBM_073	CTTTTTCAGCAGGCAATAAGC	*hmuS*^{Pg}/PG1553	Used to analyze gene expression by RT-qPCR (Śmiga et al., 2019)
PBM_074	GTCTTCCAACCAAGTCTCTC		
PBM_0838	GCCTTATTGGCTACGCTCAAG	*hmuT*/PG1554	Used to analyze gene expression by RT-qPCR (Śmiga et al., 2019)
PBM_0839	GCGTACATATATATACGACGTACAG		
PBM_0840	TAGTCGCCGGACTCATCTATC	*hmuU*/PG1555	Used to analyze gene expression by RT-qPCR (Śmiga et al., 2019)
PBM_0841	AATCTGCATCGAGGACTTCG		
PBM_0842	GCAAGGACAACATGGAAATC	*hmuV*/PG1556	Used to analyze gene expression by RT-qPCR (Śmiga et al., 2019)
PBM_0843	CCGGCACGTAGATGATTTC		
PBM_0640	TCTTCATGTGATTCCCGGCC	*ihtB*/PG0669	Used to analyze gene expression by RT-qPCR (Śmiga et al., 2024)
PBM_0641	GGTGCCGTGTCCCATGAATA		
PBM_0751	GAGTGTGGGTGCTAATGCCG	*kgp*/PG1844	Used to analyze gene expression by RT-qPCR (Śmiga et al., 2019)
PBM_0752	CACCAATATGGGTAATATTGCCG		
PBM_0753	CGCTTCCCATTCTATCACGC	*rgpA*/PG2024	Used to analyze gene expression by RT-qPCR (Śmiga et al., 2019)
PBM_0754	CGGATCTTCGTTACGCATAATCAT		
PBM_0755	AATGATAAGCCTTATACTGTAGCTG	*rgpB*/PG0506	Used to analyze gene expression by RT-qPCR (Śmiga et al., 2019)
PBM_0756	GTTTTGTGCTTCGAATACCATGC		
PBM_0771	CTATCCTGGTGGGACTGCATC	*fimA*/PG2132	Used to analyze gene expression by RT-qPCR (Śmiga and Olczak, 2019)

PBM_0772	ACCAAAGAATTGCCGAAAATC		
PBM_1501	AGTGCGAAAAGAAGGAAAGC	PG1180	Used to analyze gene expression by RT-qPCR (this study)
PBM_1502	CATTGTTCTTTCAATGTCG		
PBM_1503	CGGATTCGTGAACCTGAC	PG0217	Used to analyze gene expression by RT-qPCR (this study)
PBM_1504	GTAAGCCCCTCGACATCC		
PBM_1505	TGGATCGAAAGGCTCTGC	PG0063	Used to analyze gene expression by RT-qPCR (this study)
PBM_1506	TCTTCTCGGGCATATGCC		
PBM_1507	CAACTGCCAGACTATGCCG	*fimB*/PG2133	Used to analyze gene expression by RT-qPCR (this study)
PBM_1508	AGTAGTATCAAGTGTGGG		
PBM_1509	ATGCCGGATCATACGAGC	PG1006	Used to analyze gene expression by RT-qPCR (this study)
PBM_1510	GTACTGGATCGTATTAGC		
Synthesized DNA fragment	**AAGAAAGAGACCCGCTCGAGTGAGGATACCTCCAGCCCTGTCACCTCTTACCTGCTGCCTGCAGGAGAGCCCTTGTGGTGATAATCCTGATACTCTTCCTCATTGCCAAGCGCAGAAAAGGGAAAGAA**GGATCCAGCTATCCCTATGATGTGCCAGACTATGCTGGCTATCCATATGATGTTCCTGATTATGCTGGATACCCTTATGATGTGCCAGACTATGCCTAAGAGAAGTCCTGCTGACTATAGCCTTATTGGCTACGCTCAAGTATCTGTTCGAGGAGGGAGTACATAATTCCCTCCTCGTCCGTTGCTTGCATGCGGCGGTCTACGCGGCTTTCATCGCACTCGTACACGGACTGACGCAGCACGTCAGCCGGATGGAAGTCGAGACCCTCCTGTACTCGCCCGAAGCCCTGCGCAATGTgatccactagttctagagc	DNA fragment used for cloning	The sequence of the synthesized DNA fragment comprises: 3' region of the *hmuS*^{Pg} gene (bold capital letters), the sequence encoding 3×HA epitope (capital letters), the 3' region encoding the fragment of the *hmu* operon downstream region (capital letters underlined), and the overlapping fragment of the pTIO-1 plasmid (small letters) for cloning into the pUC57 plasmid (GenScript)
PBM_0953	gacaaggcaagggaagaacGTGTCATTGATCATACCATAAACACAC	*hmu* promoter	Amplify DNA sequence encompassing the *hmu* promoter with overlapping sequences to the pTIO-1 plasmid and the *hmuS*^{Pg} gene (indicated with small letters) (this study)
PBM_0954	cagataaattattttttcttcatAATTATCTGACCTTACTTTTAATAGGTTTCATCCG		
PBM_0955	taaaagtaaggtcagataattATGAAGAAAAAAATAATTTATCTGTCGGTAGCC	*hmuS*^{Pg}/PG1553	Amplify the majority of the *hmuS*^{Pg} gene with the overlapping sequence to the *hmu* promoter (indicated with small letters) (this study)
PBM_0956	CTCGAGCGGGTCTCTTTCTT		

PBM_0957	AAGAAAGAGACCCGCTCGAG	*hmuS^{Pg}-3×HA-DSR*	Amplify DNA sequence encompassing the 3' region of the *hmuS^{Pg}* gene, the sequence encoding 3×HA tag, and the fragment of the 3' *hmu* operon downstream region (DSR) with overlapping sequence to the pTIO-1 plasmid (indicated with small letters) (this study)
PBM_0958	gtgtcattgatcAGTGGATCACATTGC		

References

Gmiterek A, Wójtowicz H, Mackiewicz P, Radwan-Oczko M, Kantorowicz M, Chomyszyn-Gajewska M, Frąszczak M, Bielecki M, Olczak M, Olczak T (2013) The unique *hmuY* gene sequence as a specific marker of *Porphyromonas gingivalis*. PLoS ONE 8:e67719. doi:10.1371/journal.pone.0067719.

Maeda H, Fujimoto C, Haruki Y, Maeda T, Kokeguchi S, Petelin M, Arai H, Tanimoto I, Nishimura F, Takashiba S (2003) Quantitative real-time PCR using TaqMan and SYBR Green for *Actinobacillus actinomycetemcomitans*, *Porphyromonas gingivalis*, *Prevotella intermedia*, *tetQ* gene, and total bacteria. FEMS Immunol Med Microbiol 39:81-86. doi:10.1016/S0928-8244(03)00224-4.

Śmiga M, Olczak T (2019) PgRsp is a novel redox-sensing transcription regulator essential for *Porphyromonas gingivalis* virulence. Microorganisms 7:623. doi:0.3390/microorganisms7120623.

Śmiga M, Ślezak P, Olczak T (2024) Comparative analysis of *Porphyromonas gingivalis* A7436 and ATCC 33277 strains reveals differences in the expression of heme acquisition systems. Microbiol Spectr 12:e0286523. doi:10.1128/spectrum.02865-23.

Śmiga M, Ślezak P, Wagner M, Olczak T (2023) Interplay between *Porphyromonas gingivalis* hemophore-like protein HmuY and Kgp/RgpA gingipains plays a superior role in heme supply. Microbiol Spectr 11:e0459322. doi:10.1128/spectrum.04593-22.

Śmiga M, Stępień P, Olczak M, Olczak T (2019) PgFur participates differentially in expression of virulence factors in more virulent A7436 and less virulent ATCC 33277 *Porphyromonas gingivalis* strains. BMC Microbiol 19:127. doi:10.1186/s12866-019-1511-x.

Tagawa J, Inoue T, Naito M, Sato K, Kuwahara T, Nakayama M, Nakayama K, Yamashiro T, Ohara N (2014) Development of a novel plasmid vector pTIO-1 adapted for electrotransformation of *Porphyromonas gingivalis*. J Microbiol Methods 105:174-179. doi:10.1016/j.mimet.2014.07.032.
